# EARLY BUD-BREAK 1 and EARLY BUD-BREAK 3 control resumption of poplar growth after winter dormancy

**DOI:** 10.1038/s41467-021-21449-0

**Published:** 2021-02-18

**Authors:** Abdul Azeez, Yiru Chen Zhao, Rajesh Kumar Singh, Yordan S. Yordanov, Madhumita Dash, Pal Miskolczi, Katja Stojkovič, Steve H. Strauss, Rishikesh P. Bhalerao, Victor B. Busov

**Affiliations:** 1 College of Forest Resources and Environmental Science, Michigan Technological University, Houghton, MI USA; 2 Umeå Plant Science Centre, Department of Forest Genetics and Plant Physiology, Swedish University of Agricultural Sciences, Umeå, Sweden; 3 Department of Biotechnology, CSIR-Institute of Himalayan Bioresource Technology, Palampur, Himachal Pradesh India; 4 Department of Biological Sciences, Eastern Illinois University, Charleston, IL USA; 5 Department of Forest Ecosystems and Society, Oregon State University, Corvallis, OR USA

**Keywords:** Agricultural genetics, Plant development, Plant molecular biology, Plant signalling

## Abstract

Bud-break is an economically and environmentally important process in trees and shrubs from boreal and temperate latitudes, but its molecular mechanisms are poorly understood. Here, we show that two previously reported transcription factors, EARLY BUD BREAK 1 (EBB1) and SHORT VEGETATIVE PHASE-Like (SVL) directly interact to control bud-break. EBB1 is a positive regulator of bud-break, whereas SVL is a negative regulator of bud-break. EBB1 directly and negatively regulates *SVL* expression. We further report the identification and characterization of the EBB3 gene. EBB3 is a temperature-responsive, epigenetically-regulated, positive regulator of bud-break that provides a direct link to activation of the cell cycle during bud-break. EBB3 is an AP2/ERF transcription factor that positively and directly regulates *CYCLIND3.1* gene. Our results reveal the architecture of a putative regulatory module that links temperature-mediated control of bud-break with activation of cell cycle.

## Abstract (web summary)

An AP2/ERF family gene *EBB1* and a MADS-box gene *SVL* encode two regulators of poplar bud break. Here, the authors report another AP2/ERF transcription factor EBB3, which functions together with EBB1, SVL, and cell cycle progression promoter CYCD3.1 to regulate poplar bud break.

## Introduction

The alteration of periods of active growth and dormancy is a widespread adaptive strategy in plants from seasonal climates that enable them to survive unfavorable conditions associated with prolonged periods of low temperature and/or moisture stress. In boreal and temperate woody perennials, winter bud dormancy is developed in the fall and involves, in chronological order: cessation of shoot elongation, the formation of buds (bud-set), and establishment of dormancy. In most woody plants, including *Populus*, cessation of shoot growth and the induction of dormancy are either induced or accelerated by short days (SDs), and prevented or delayed by long days (LDs)^1,2^. The integration of photoperiod detection and growth inhibition involves the convergence of the regulatory activities of the circadian clock machinery (involved in photoperiod sensing) on the *FT (FLOWERING LOCUS T)* regulatory hub^3–6^. In poplar, the clock daylength/nightlength sensing components LHY1 (LATE ELONGATED HYPOCOTYL 1), LHY2, GI (GIGANTEA), and CO1/2 (CONSTANS) regulate *FT2* (one of two FT paralogs) expression in accordance with the day length^4,7–9^. High *FT2* expression promotes active growth, while *FT2* repression leads to early growth cessation and bud set^6,10^. The signaling downstream of FT2 involves Like-APETALA1 (LAP1), which directly and positively regulates *AINTEGUMENTA-Like 1* (*AIL1*) gene^11^, while AIL1 directly regulates D-type cyclins, an important cell cycle progression check point^12^. Thus, growth cessation and bud-set prior to dormancy establishment have co-opted genes and signaling cascades that regulate the photoperiodic floral initiation pathway.

Following growth cessation and bud set, continual exposure to SD results in the establishment of a dormant state during which buds are insensitive to growth-promoting signals^13^. It was recently shown that abscisic acid (ABA) plays a major role in the establishment of bud dormancy^14^. Specifically, under SDs, ABA concentration and signaling increase and promote the biosynthesis and deposition of callose at the plasmodesmata (PD) to develop obstructions known as PD sphincters^15–18^. These symplastic blockages isolate shoot apical meristem (SAM) from growth-promoting signals^14,19^.

Once dormancy is established, resumption of active growth requires prolonged exposure of the bud to low temperatures^5,20–22^. This phase, known as dormancy release, ensures that plants resume growth only after the stable return of favorable growth conditions; in essence, it represents a clock measuring the length of winter^23^. Although both LDs and warm temperatures are required for a return to active growth, the dominant triggering signal is warm temperatures^23^. Therefore, in contrast to growth cessation and dormancy establishment phases, which are photoperiod-regulated processes, dormancy release and reactivation of growth are primarily thermo-regulated processes^24,25^. How temperature controls dormancy release and bud-break is poorly understood at the molecular level.

The discovery of the *Dormancy Associated MADS-box* (*DAM*) genes from the evergreen peach mutant has led to speculation that dormancy release and bud-break may share similarities with vernalization^26^. Indeed, *DAM* genes and *MADS-box* genes like *FLOWERING LOCUS C* (*FLC*) are repressed during vernalization, which by low temperatures, and expression changes are correlated with changes in activating/repressive histone modifications^27,28^. Furthermore, over-expression of *DAM* genes causes delayed bud-break similar to how *FLC* overexpression leads to delayed flowering^29–31^. However, since evergreen mutant does not cease growth in response to SDs, its relevance to dormancy and bud-break remains unclear. Similarly, co-suppression of an apple *DAM1* (*MdDAM1*) led to an inability of the transgenic plants to cease growth and establish dormancy^32^. Recently, another *MADS-box* gene with homology to *SVP* in *Arabidopsis*, named *SVP-like* (*SVL*), was shown to negatively regulate bud-break^29^. SVL not only regulates bud-break but is also a major hub in the signaling cascade leading to dormancy, downstream of ABA signaling^29^. Expression of *SVL* is regulated by both SD photoperiod during dormancy initiation and low temperature during dormancy release^29,33^. Thus, SVL provides the regulatory link between the photoperiodic and thermo-signaling pathways during the onset of dormancy and its release.

Earlier we identified the poplar *EARLY BUD-BREAK 1* (*EBB1*) gene encoding a transcription factor of the AP2/ERF family with high homology to *Arabidopsis* SAM activity regulator DORNROSCHEN (DRN), as a conserved positive regulator of bud-break^34,35^. However, the regulatory context of EBB1 and its link with the SVL pathway remained unclear. As a result, there are significant gaps in our knowledge of how bud-break is regulated. Here, we demonstrate that EBB1 acts as a direct upstream repressor of SVL. Importantly, we identify EBB3, a transcription factor of the AP2/ERF family as a component of the EBB1-SVL bud-break pathway. Our results demonstrate that EBB3 is epigenetically regulated by low temperature and promotes bud-break by positively regulating cell proliferation-related genes. The elucidation of the role of EBB3 thus provides a missing link of low-temperature signals, cell proliferation, and the control of bud-break.

## Results

### *Early bud-break 3D* mutant discovery

**Fig. 1:**
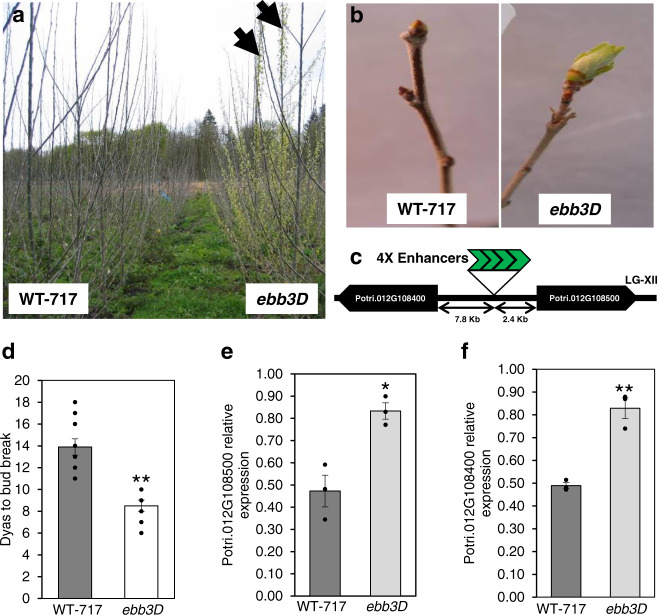
Isolation and molecular analysis of the *early bud-break 3D* (*ebb3D*) poplar mutant. **a** *ebb3D* poplar mutant plants show early bud-break in the field during the start of the second growing season as compared to neighboring WT-717 control trees, arrows point to two *ebb3D* ramets that show early bud-break compared to the WT-717 plants and other neighboring activation tagging events. **b** Early bud-break in *ebb3D* mutant plant as compared to WT-717 under controlled growth chamber conditions (see Methods for more details). **c** Genome position of the activation tag insertion in the *ebb3D* mutant, 4X enhancers derived from the CaMV35S promoter. **d** The average number of days to bud-break in WT-717 and *ebb3D* mutant plants. **e**, **f** Potri.012G108400 and Potri.012G108500 genes are upregulated in the *ebb3D* mutant. Expression values are the average of three biological replicates ±SEM, normalized to the reference *ACT7* gene. At least 10 plants of the *ebb3D* mutant and WT-717 genotypes were used in the bud-break analysis. Asterisks (*) and (**) indicate significant differences at *P* < 0.02 and *P* < 0.002 compared to WT-717 control plants by two-tailed paired *t*-tests. Source data underlying Fig. 1a–d are provided as a Source Data file.

A population of activation tagged WT-717 (*Populus tremula X Populus alba*) was screened in a field trial and a mutant with early bud-break was identified (Fig. 1a). The early bud-break phenotype of the mutant was also validated under controlled growth chamber conditions. Similar to our field observation, mutant plants showed precocious bud-break that was around 6 days earlier as compared to WT-717 (Fig. 1b, d). Because of the early bud-break phenotype, the mutant was named *early bud-break 3 Dominant* (*ebb3D*) and the corresponding gene *EBB3* (*EARLY BUD-BREAK 3*). We positioned the tag in the genome sequence on chromosome XII and found two genes (Potri.012G108400 and Potri.012G108500) flanking the insertion within 10Kb up and downstream of the insertion site. Potri.012G108400 was 7.8Kb upstream and Potri.012G108500 2.4Kb downstream of the activation tag insertion (Fig. 1c). Next, we compared the expression of these two genes in the *ebb3D* mutant and WT-717 plants. Both genes were upregulated in *ebb3D* mutants as compared to WT-717 plants (Fig. 1e, f). Potri.012G108400 encodes a protein similar to ribosomal protein L34e (RPL34e), which is involved in translation and ribosome biogenesis. Potri.012G108500 encodes an AP2/ERF domain-containing transcription factor with similarity to PtERF113 of subfamily B4 (Supplementary Fig. 1).

### EBB3 is PtERF113 and its overexpression leads to early bud-break while its suppression causes a delay in bud-break

**Fig. 2:**
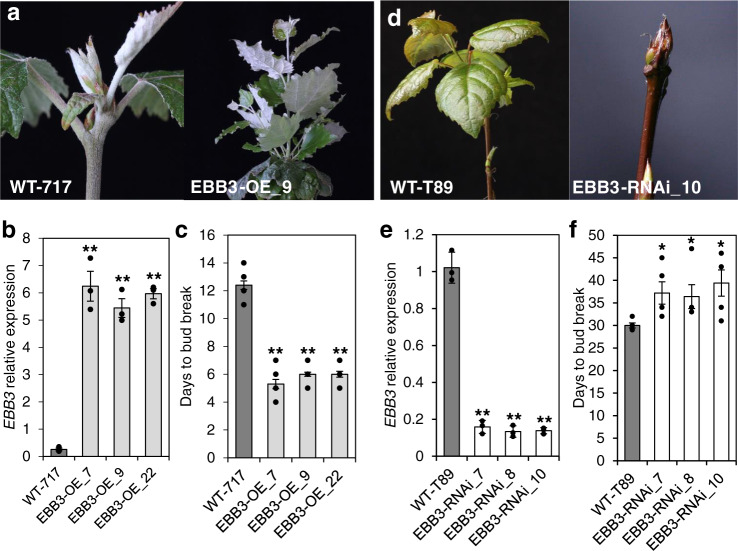
EBB3 over- and under-expressing lines show early and late bud-break phenotypes respectively. **a** Early bud-break of a representative over-expressing line compared to WT-717 control plant. **b** *EBB3* relative expression in EBB3-OE lines. **c** Time to bud-break in WT-717 plants and EBB3-OE lines. **d** Delayed bud-break in representative EBB3 under-expressing line compared to WT-T89 control plant. **e** *EBB3* relative expression in EBB3-RNAi lines. **f** Time to bud-break relative to WT-T89 control plants in EBB3-RNAi lines. Expression values are average of three biological replicates ±SEM, normalized to the reference *ACT7* for WT-717 and *UBQ* gene for WT-T89 clones, respectively. Asterisks (*) indicate significant and (**) indicate extremely significant differences compared to WT at *P* < 0.05 and *P* < 0.0001, respectively, and determined by two-tailed paired *t*-tests. Source data underlying Fig. 2a, c, and d are provided as a Source Data file.

Since both candidate genes were activated in *ebb3D* mutant we proceeded to pinpoint the causal gene underlying the phenotype by overexpressing them in the parental WT-717 genetic background. A total of 13 Potri.012G108400/RPL34e and 18 Potri.012G108500/PtERF113 overexpressing independent transgenic events were recovered and three lines with high overexpression (Fig. 2b and Supplementary Fig. 2a) of the two transgenes were tested for the *ebb3D* phenotype. The overexpression transgenics for Potri.012G108400/RPL34e showed no phenotypic differences compared to WT-717, including the timing of bud-break (Supplementary Fig. 2b). In contrast, all three Potri.012G108500 /PtERF113-overexpressing lines (EBB3-OE_7, EBB3-OE_9, and EBB3-OE_22) showed significantly early bud-break as compared to WT-717 control plants (Fig. 2a–c). Therefore, the gene responsible for the *ebb3D* phenotype is PtERF113, hereafter, referred to as Early Bud-break 3 (EBB3), and transgenic lines overexpressing this gene as EBB3-OE. To further confirm the function of EBB3 in bud-break, we downregulated the expression of *EBB3* (EBB3-RNAi). Three independent transgenic lines (EBB3-RNAi) with significantly reduced *EBB3* expression were generated. Contrary to EBB3 overexpressers, all three EBB3-RNAi lines (EBB3-RNAi_7, EBB3-RNAi_8, and EBB3-RNAi_10) showed significantly delayed bud-break compared to WT-T89 (Fig. 2d–f). Taken together, these results show that EBB3 (PtERF113) is a positive regulator of bud-break in poplar.

### *EBB3* expression pattern further supports its role in the control of bud-break

**Fig. 3:**
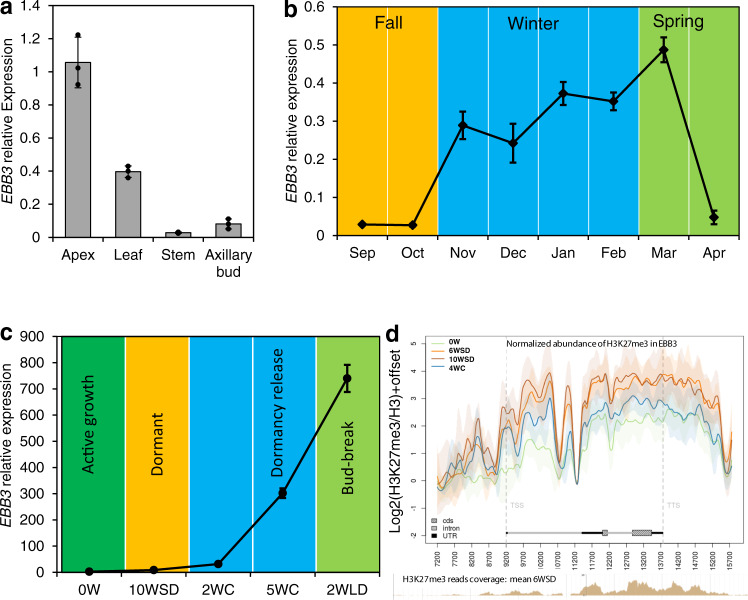
*EBB3* is primarily expressed in the shoot apex and differentially regulated during the dormancy cycle and bud-break. **a** Relative expression of *EBB3* in different tissues of WT-717 plants. **b** Relative expression of *EBB3* in vegetative buds of wild-growing aspen (*Populus tremuloides*) trees. **c** Relative expression of *EBB3* in WT-T89 plants under manipulative dormancy treatments in controlled growth chamber conditions. Expression values are the average of three biological replicates ±SEM, normalized to the reference genes *ACT7* for WT-717 clones, and *UBQ* for WT-T89 clones, respectively. 0 W: time point before inductive treatments, 10 WSD: 10 weeks under SD photoperiod, 2WC: 2 weeks under cold, 5WC: 5 weeks under cold, 2WLD: 2 weeks under LD photoperiod and warm temperature. **d** Average H3K27me3 abundance of three biological replicates in the *EBB3* locus, including 2 Kb downstream and upstream regions, before dormancy inductive treatments (0 W) and during dormancy induction, (6WSD, 10WSD) and dormancy release (4WC). H3K27me3 abundance was normalized relative to H3 abundance. Shaded areas indicate ±standard deviation. TSS transcription start site, TTS transcription termination site, cds coding sequence, UTR untranslated region. Source data underlying Fig. 3c are provided as a Source Data file.

To better understand the role of EBB3 in the regulation of dormancy we studied its expression pattern during active growth and through a dormancy cycle. *EBB3* is highly and primarily expressed in the actively growing shoot apex (Fig. 3a). We first studied the expression of the gene in wild aspen trees in the field during the activity-dormancy cycle. *EBB3* expression was very low during growth cessation and dormancy establishment (Sep–Oct) and highly upregulated during winter/spring months (Nov-March) (Fig. 3b). This suggests that *EBB3* expression may be regulated by low-temperature signals. Consistent with the role of EBB3 as a positive regulator of bud-break, its expression peaks right before or at the time of bud-break in March. Interestingly, *EBB3* was precipitously downregulated post-bud-break in April, suggesting a specific functional role in bud-break (Fig. 3b). To more precisely correlate the expression of *EBB3* with different stages of the activity-dormancy cycle in the natural condition, we exposed poplar plants (WT- T89) to a regime of SDs (10WSD: 10 weeks under SD photoperiod) to induce growth cessation and dormancy, followed by exposure to 5 weeks of cold temperatures (4 °C) (5WC) (dormancy release) and 2 weeks of a LD and warm temperature (2WLD) to induce bud-break. *EBB3* expression was low in actively growing apices (0 W: time point before inductive treatments) and under SD photoperiod (10WSD) (Fig. 3c). *EBB3* was strongly upregulated after 2 weeks of exposure to low temperature (2WC), remained high after 5 weeks of cold treatment (5WC), and further increased following bud exposure to warm temperature and LD (bud-break) (2WLD) (Fig. 3c). This indicates that low temperature, which is required for release from dormancy, positively regulates *EBB3* expression.

### Low-temperature induction of *EBB3* is epigenetically regulated

We then investigated how dormancy release cues such as low temperature could mediate *EBB3* expression change. Low temperatures have been found to trigger histone modifications that promote or repress transcription of regulatory genes^36^. It has been previously shown that repressive H3 lysine 27 trimethylation (H3K27me3) histone marks are responsive to cold temperature in peach and pear^27,37^. Therefore, we investigated whether *EBB3* activation after cold exposure involves changes in H3K27me3. Indeed, the repressive mark H3K27me3 was significantly decreased at the *EBB3* locus after exposure to low temperature that promotes dormancy release (Fig. 3d). The temporal correlation between the decrease in H3K27me3 repressive marks in the *EBB3* gene and its upregulation after low-temperature exposure (Fig. 3c) strongly suggests that H3K27me3 modification plays role in *EBB3* regulation during dormancy release.

### EBB1 directly suppresses *SVL* expression

**Fig. 4:**
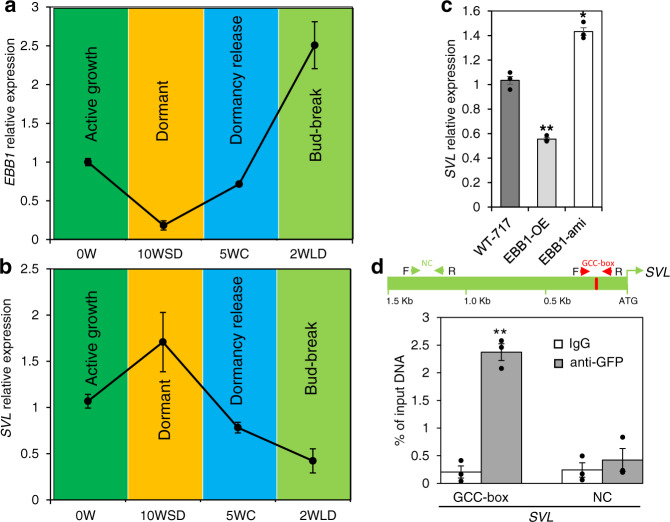
EBB1 directly binds to *SVL* promoter in vivo in chromatin immunoprecipitation (ChIP) assays to regulate its expression. **a** *EBB1* and **b** *SVL* relative expression during dormancy cycle and bud-break under manipulative dormancy treatments in controlled growth chamber conditions; 0 W: time point before inductive treatments, 10WSD: 10 weeks under SD photoperiod, 5WC: 5 weeks under cold, 2WLD: 2 weeks under LD photoperiod and warm temperature. **c** Relative expression of *SVL* in EBB1 over-expressing (EBB1-OE) and underexpressed (EBB1-ami) plants. Expression values are the average of three biological replicates ±SEM, the expression values normalized to the reference *ACT7* gene. **d** Enrichment of a DNA fragment in the *SVL* promoter containing a GCC-box and quantified by ChIP-qPCR. The green box is a schematic representation of the *SVL* promoter showing the position of the GCC-box (red). Red arrows delineate the position of DNA fragments containing the GCC-box and green arrows demarcate the position of DNA fragments with no GCC-box used as a negative control (NC) in ChIP-qPCR analysis. Chromatin from EBB1-GFP DNA transfected poplar protoplasts was isolated using anti-GFP antibody and IgG used as a control antibody. ChIP-purified DNA was used to perform ChIP-qPCR, expression values are represented as the percentage of input (% of input) DNA. Values are the average of three biological replicates ±SEM. Asterisks (*) and (**) indicate extreme significant differences compared to their respective control at *P* < 0.003 and *P* < 0.0005 respectively and determined using two-tailed paired multiple *t*-tests. Source data underlying Fig. 4d are provided as a Source Data file.

To better understand how the regulatory roles of EBB1, SVL, and EBB3 (the three known genes that control bud-break) are integrated, we first investigated potential interactions between EBB1 and SVL. We studied the *EBB1* and *SVL* expression during the active growth and dormancy cycle under a controlled environment. *EBB1* expression was downregulated under SD photoperiod (10WSD) (Fig. 4a), strongly upregulated after exposure to low temperature (dormancy release) (5WC), and further increased following exposure of the bud to warm temperatures and LDs (bud-break) (2WLD) (Fig. 4a). The pattern of *SVL* expression was opposite to that for *EBB1*. *SVL* expression was relatively high during SDs, repressed by low temperatures and remained low during warm temperatures and LDs (Fig. 4b). Since the expression profiles of these two genes indicated that they may regulate each other, we studied the expression of *SVL* in EBB1 over- (EBB1-OE) and under-expressing (EBB1-ami) plants. *SVL* expression was significantly repressed in EBB1 overexpressers and upregulated in EBB1 downregulated plants (Fig. 4c). These results indicate that EBB1 may act upstream of *SVL* to repress its expression. To ascertain if EBB1 directly regulates *SVL*, we employed EMSAs (Electrophoretic Mobility Shift Essays) to determine if EBB1 binds to the *SVL* promoter. Since EBB1 is an AP2/ERF transcription factor, binds to a consensus motif (GCCGCCA) known as a GCC-box^38^, we scanned the −2000 bp putative *SVL* promoter region and found a GCC-box −150 bp from the translation start site (Fig. 4d). EBB1 bound specifically to the GCC-box (27 bp); no binding occurred with three mutated versions of the GCC-Box (Details in “Methods”, Supplementary Fig. 3). These data indicate that EBB1 directly binds to the *SVL* promoter under in vitro conditions. Further, to confirm in vivo binding of EBB1 to *SVL* promoter we performed ChIP-qPCR (Chromatin immunoprecipitation-quantitative PCR). Chromatin was isolated from EBB1-GFP DNA transfected poplar protoplasts using an anti-GFP antibody, whereas the IgG antibody was used as control. Consistent with the in vitro EMSA binding assay, the ChIP-qPCR results showed strong binding of EBB1 with the regulatory region of the *SVL* containing the GCC-box (Fig. 4d). Thus, the EMSA and ChIP-qPCR results indicate that EBB1 directly binds to the *SVL* promoter to regulate its expression.

### EBB3 is downstream of EBB1 and SVL

**Fig. 5:**
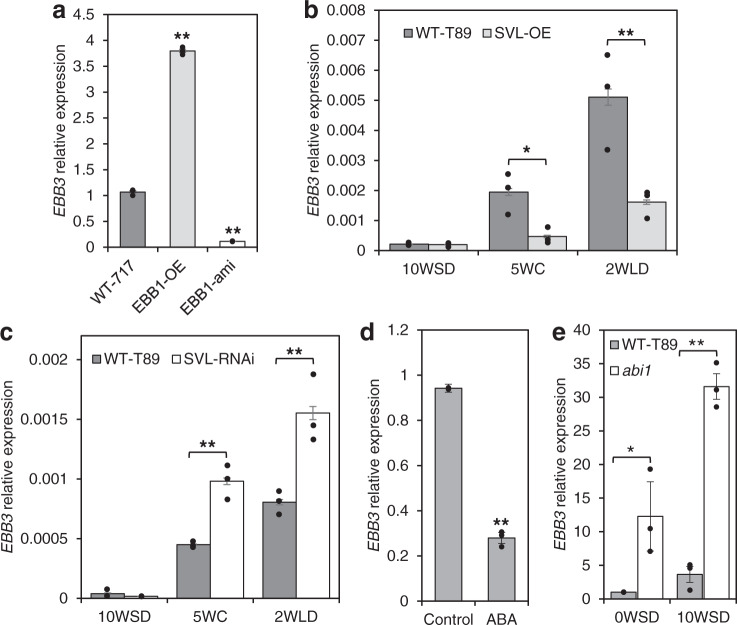
EBB1, SVL, and ABA regulate the expression of *EBB3*. **a** Relative expression of *EBB3* in EBB1 over-expressing and under-expressing plants. Relative expression of *EBB3* in SVL over-expressing (**b**) and under-expressing (**c**) plants. **d** Relative expression of *EBB3* in ABA-treated apices of WT-717 plants. **e** *EBB3* expression in apices of transgenic hybrid aspen with reduced ABA response (*abi1* mutant); 0W: time point before inductive treatments, 10WSD: 10 weeks under SD photoperiod, 5WC: 5 weeks under cold, 2WLD: 2 weeks under LD photoperiod and warm temperature. Expression values are the average of three biological replicates ±SEM, normalized to the reference genes - *UBQ* for WT-T89 and *ACT7* for WT-717 clones respectively. Asterisks (*) indicate significant and (**) indicate extremely significant differences compared to WT at *P* < 0.04 and *P* < 0.001 respectively and determined using two-tailed paired multiple *t*-tests.

To determine if EBB3 is part of the EBB1/SVL-mediated regulatory mechanism, we first examined the expression of *EBB3* in EBB1 transgenic plants. *EBB3* was significantly upregulated in EBB1-OE plants and downregulated in EBB1-ami plants (Fig. 5a). These data indicate that EBB1, EBB3, and SVL are part of a common regulatory mechanism and that EBB1 acts upstream of SVL and EBB3 to regulate their expression. To determine whether EBB3 acts up- or downstream of SVL, we studied *SVL* expression in EBB3 overexpressing (EBB3-OE) and downregulated (EBB3-RNAi) transgenic plants. There was no significant change in the expression of *SVL* in EBB3-OE or EBB3-RNAi plants (Supplementary Fig. 4a, b). We also performed EMSAs using HA-tagged EBB3 protein and putative binding sites in the *SVL* promoter, HA-EBB3 does not bind to the *SVL* promoter (Supplementary Fig. 4c). Further, we also checked in vivo binding of EBB3 to *SVL* promoter by ChIP-qPCR assay. Consistent with our in vitro binding assay, no evidence of EBB3 binding to the regulatory region of the *SVL* was detected (Supplementary Fig. 4d). These results suggest that EBB3 is downstream of SVL with respect to the regulation of bud-break. To confirm this, we investigated *EBB3* expression in *SVL* transgenics. *EBB3* was downregulated in *SVL* overexpressing (SVL-OE) plants whereas conversely it was upregulated in *SVL* suppressed (SVL-RNAi) plants (Fig. 5b, c). These results further confirmed that EBB3 acts downstream of SVL.

### Bud-break suppressor ABA negatively regulates EBB3

It was recently shown that ABA is a major regulator of bud dormancy and repressor of bud-break^14^. ABA also positively regulates *SVL* expression, while SVL in turn positively regulates ABA biosynthesis and signaling-related genes thus forming positive feedforward loop^29^. Therefore, we investigated whether ABA regulates *EBB3* expression. Indeed, ABA treatment significantly downregulated *EBB3* expression in shoot apices (Fig. 5d). Furthermore, *EBB3* expression in transgenic hybrid aspen with a reduced ABA response (*abi1-1*) was significantly higher than in WT-T89 plants (Fig. 5e). Because GA biosynthesis is upregulated in the *abi1-1* poplar mutant^14^, we wanted to differentiate whether the elevated *EBB3* expression in *abi1-1* transgenics is a result of the reduced ABA response or increased GA levels. We, therefore, studied *EBB3* expression in GA-treated, WT-717 shoot apices. No significant changes in *EBB3* expression were found after GA treatment (Supplementary Fig. 5). Thus, these data indicate that EBB3 is a target of ABA signaling downstream of the SVL/ABA feedforward loop during dormancy release and bud-break.

### Extensive transcriptomic changes are associated with modulation of *EBB3* expression during the activity-dormancy cycle

To better understand the EBB3 mediated control of bud-break and to identify the downstream target of EBB3 involved in bud-break, we performed transcriptome analysis of EBB3-RNAi transgenics during the activity-dormancy cycle. We focused on apices because of the highest expression of the gene in these tissues. There were 29 differentially expressed genes after dormancy establishment (10WSD), 110 after 5-week cold treatment (5WC), and 1162 at the time of bud-break (2WLD) (Supplementary Data 1–7, Supplementary Table 2, see also “Methods” for statistical analysis). The number of differentially expressed genes in the different dormancy stages corresponds well with the native *EBB3* expression level during dormancy release and bud-break.

### EBB3 directly regulates *CYCD3.1* gene expression

**Fig. 6:**
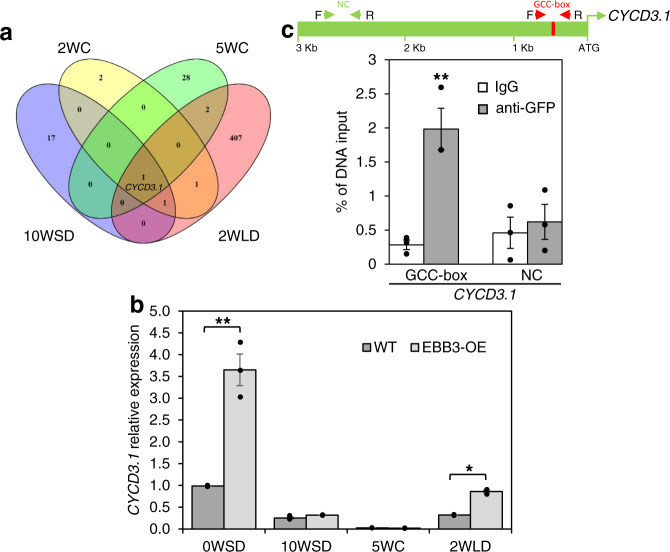
EBB3 binds directly to the *CYCD3.1* promoter in vivo in chromatin immunoprecipitation assays to regulate its expression. **a** Number of common and unique downregulated genes in EBB3 RNAi under-expressing plants in different dormancy stages. The *Cyclin D3.1* (*CYCD3.1*) is the only downregulated gene in all dormancy stages. See Supplementary Data 4–7 for more details. **b** Relative expression of *CYCD3.1* in EBB3-OE and WT-717 plants; 0W: time point before inductive treatments, 10WSD: 10 weeks under SD photoperiod, 5WC: 5 weeks under cold, 2WLD: 2 weeks under LD photoperiod and warm temperature, expression values are normalized to the *ACT7* gene. Expression values are the average of three biological replicates ±SEM. **c** Enrichment of a DNA fragment in the *CYCD3.1* promoter containing a GCC-box and quantified by ChIP-qPCR. The green box is a schematic representation of the *CYCD3.1* promoter showing the position of the GCC-box (red). Red arrows delineate the position of DNA fragments containing the GCC-box and green arrows demarcate the position of DNA fragments with no GCC-box used as a negative control (NC) in ChIP-qPCR analysis. Chromatin from EBB3-GFP DNA transfected poplar protoplasts was isolated using anti-GFP antibody and IgG used as a control antibody. ChIP-purified DNA was used to perform ChIP-qPCR, expression values are represented as percentage input (% input) DNA. Values are the average of three biological replicates ±SEM. Asterisks (*) and (**) indicate significant differences compared to their respective control at *P* < 0.05 and *P* < 0.004 respectively and determined using two-tailed paired multiple *t*-tests. Source data underlying Fig. 6a and c are provided as a Source Data file.

The *CYCD3.1* (Potra002502g18897) gene was the only gene among all differentially regulated genes, that was statistically significantly (*P* < 0.05) downregulated in the EBB3-RNAi plants at all time points studied using RNA-seq (Fig. 6a, Supplementary Data 4–7). We, therefore, studied the expression of the *CYCD3.1* gene in EBB3-OE plants using qRT-PCR and found that in contrast to EBB3-RNAi plants where the gene was significantly downregulated, *CYCD3.1* was significantly upregulated in EBB3-OE plants (Fig. 6b). These data further suggested that *CYCD3.1* is downstream of EBB3. EBB3 is an AP2/ERF transcription factor that binds to a consensus GCC-box^38^, and we identified a GCC-box-like (GCCGGGCCA) motif in the promoter region of *CYCD3.1*. To confirm that *CYCD3.1* is a direct target of EBB3, we performed EMSA using 6xHIS-tagged EBB3-purified protein and the 27 bp GCC-box. The EBB3 protein specifically bound the GCC-box fragment of the *CYCD3.1* promoter, whereas no binding was observed with the mutated version of the GCC-Box (TCCTTTCCA) (Supplementary Fig. 6). Further, we checked in planta EBB3 binding to the GCC-box of CYCD3.1 promoter using ChIP-qPCR assays in poplar protoplasts. EBB3 showed clear binding to the part of *CYCD3.1* promoter containing the GCC-box (Fig. 6c). These results show that EBB3 positively and directly regulates *CYCD3.1* expression during dormancy release and bud-break.

## Discussion

**Fig. 7:**
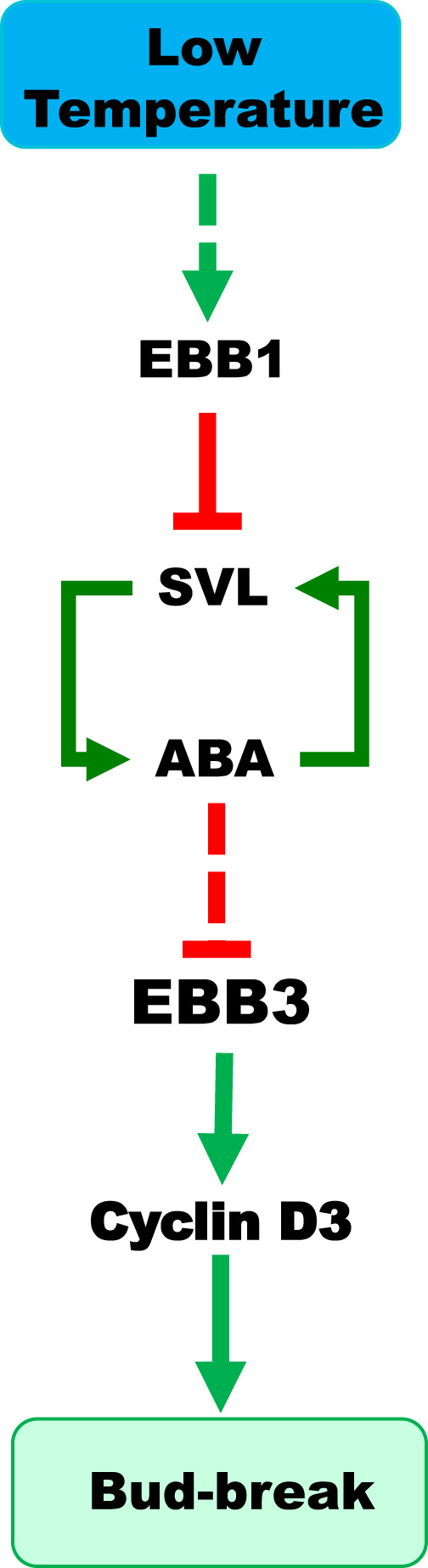
Hypothetical model of the roles EBB1, SVL and EBB3 play in control of bud-break in poplar. EBB1 is positively regulated by low temperature, leading to suppression of *SVL* expression. Declining *SVL* expression breaks the SVL/ABA feedforward loop. SVL/ABA repression leads to the upregulation of *EBB3* and consequently to activation of *CYCD3.1*, cell proliferation, and bud-break. Green arrows indicate positive regulation while red bars indicate negative regulation. Dash arrows indicate indirect regulation. See the text for additional description.

We report the identification of a putative regulatory pathway comprised of three transcription factors (EBB1, SVL, and EBB3) that mediate bud-break in poplar (Fig. 7). Our gene expression data, in vivo/in vitro binding assays, and analysis of gain- and loss-of-function transgenics indicate that EBB1 acts at the top of the pathway and represses the expression of its direct downstream target *SVL*. Our data clearly show that hierarchically, SVL/ABA reinforcing loop acts upstream of EBB3 and negatively regulates its expression. Downstream, EBB3 directly and positively regulate *CYCLIND3.1* (*CYCD3.1*), a key promoter of the G1/S progression of the cell cycle whose expression correlates with bud reactivation of growth at bud-break^20,39^. The identification of these regulatory interactions would benefit further from in planta analysis of their spatiotemporal specificity during dormancy-activity cycle. Furthermore, genetic evidence derived from double and triple mutants is needed to confirm the functionality and hierarchy of the proposed model.

While EBB1 and SVL were previously discovered as regulators of bud-break^29,34^, here we elucidated the interaction between these two components of the bud-break pathway by showing that EBB1 directly and negatively regulates *SVL* expression. This regulation is likely most relevant at the activity-dormancy transitions as suggested by the dramatic and opposing changes in the expression of these two genes that occur at these transition periods. *EBB1* is downregulated, while *SVL* is upregulated during dormancy induction and establishment (Fig. 4). Thus, *SVL* upregulation during dormancy is, at least in part, due to de-repression brought about by declining levels of *EBB1*. Previous work has shown that *SVL* is also highly ABA responsive and that ABA does increase during dormancy establishment^14,16,18,29^. Thus, *SVL* upregulation is a result of the combined and opposing regulatory activities of EBB1 and ABA. This situation is gradually reversed during dormancy release and bud-break. *EBB1* is upregulated while *SVL* is downregulated, suggesting that EBB1 after cold treatment re-establishes *SVL* repression, and this at least in part restores shoot apex competence for growth (Fig. 4). SVL and ABA form a feedforward loop; SVL upregulates expression of *NCED3* (a critical ABA biosynthetic gene), while ABA upregulates expression of *SVL*^29^. Rising EBB1 levels during dormancy release likely break this feedforward loop by downregulating the expression of *SVL* and consequently ABA levels. Thus, EBB1 not only affects *SVL* expression but also likely indirectly plays a part in reducing the concentration of ABA during chilling to release apices from dormancy.

We also report the identification and characterization of EBB3, a component of the EBB1-SVL-mediated regulatory mechanism, that controls bud-break. We present several lines of evidence that clearly implicate EBB3 in the regulation of bud-break. First, transgenic up and downregulation of the gene leads to accelerated and delayed bud-break of the transgenic plants compared to WT controls (Fig. 2). *EBB3* expression highly correlates with dormancy release and bud-break. *EBB3* is almost undetectable during dormancy establishment and significantly increases with the progression of the cold treatment peaking right around the bud-break time.

EBB3 encodes a transcription factor of the AP2/ERF family with highest homology to AtERF114, known as ERF BUD ENHANCER (EBE)^40^. In *Arabidopsis*, EBE is a positive regulator of axillary bud outgrowth^40^. Apical dominance plays a central role in axillary bud dormancy in *Arabidopsis*^41^. In contrast, winter bud dormancy is imposed by environmental cues and requires resetting through a cold treatment^23^. Thus, it is likely that dormancy regulation in the axillary and apical bud may be different, particularly upstream of EBB3/EBE. Nevertheless, the downstream processes controlled by the two genes appear to share significant similarities. For example, in *Arabidopsis*, one of the most prominent transcriptomic changes in response to inducible EBE upregulation involves cell cycle genes, including CYCDs^40^. Thus, both EBE and EBB3 appear to activate cell cycle. In *Arabidopsis*, the genes differentially regulated by EBE have an overrepresentation of TCP18/BRC1 binding sites^40^. It was previously found that in poplar, SVL positively regulates *TCP18/BRC1* during dormancy. In *Arabidopsis*, Class II TCPs, to which BRC1 belongs, repress organ growth by inhibiting cell proliferation at the G1-to-S transition^42,43^. Here we find that SVL negatively regulates *EBB3*. Thus, the increasing and decreasing abundance of SVL during dormancy can act in balancing cell proliferation and bud-break competence by regulating *TCP18/BRC1* and *EBB3*. Higher TCP18/BRC1 levels may inhibit cell proliferation and negatively regulate bud-break^44^, while high EBB3 levels post cold treatment would activate cell cycle and bud-break.

Although temperature cues have been known to regulate dormancy release and subsequent bud-break, how temperature controls these transitions has not been entirely clear. Interestingly, epigenetic changes at *EBB3* locus correlate with gene expression and exposure to dormancy releasing low-temperature signal. Our data show that following exposure to low temperature, there is a reduction in the repressive H3 lysine 27 trimethylation (H3K27me3) marks across the whole sequence of the *EBB3* gene with simultaneous upregulation of *EBB3* (Fig. 3d). Low temperatures have been found to trigger histone modification which promotes or represses transcription during vernalization and bud dormancy release^36^. For example, H3 lysine 27 trimethylation (H3K27me3) histone marks in pear *EBB1* and peach *DAM* genes are responsive to cold temperature^27,37^. This correlation between repressive marks and expression is not perfect and indicates that EBB3 is under complex regulatory mechanism including but not limited to epigenetic modifications. For example, transcription activators may upregulate the gene prior and at bud-break but repressors downregulate the gene past bud-break. Thus, the summary of all data strongly implicate EBB3 in the regulation of bud-break in poplar and this may involve at least in part low temperature-responsive changes in *EBB3* expression via epigenetic changes. The importance of epigenetic changes in the regulation of low-temperature response and bud-break was also recently demonstrated by the role of a poplar DEMETER-LIKE 10 (DML), a DNA demethylase, plays in the regulation of bud-break^45^. To find potential commonalities in the mechanisms governed by EBB3 and DML during bud-break, we compared the differentially expressed genes (DEGs) in EBB3 and DML knocked-down transgenics. A total of 106 common genes were discovered (Supplementary Data 8). Analysis of the putative function of the common genes shows gene ontology (GO) terms enrichment (FDR < 0.05) of genes involved in various metabolic processes (Supplementary Data 9) but does not show enrichment of genes involved in regulation of cell cycle or growth. This is not surprising as the two genes have very different regulatory activities and likely target different processes during bud-break.

Although here we describe regulatory interactions involving mainly transcription control, it is clear that there are multiple diverse regulatory layers that differentially act on the different components of the cascade. For example, low temperatures affect the expression of all three genes (EBB1, SVL, and EBB3). In *Arabidopsis*, low-temperature signaling is associated with changes in repressive and activation histone marks at MADS-box genes like *SVP*, which induces flowering after cold^46,47^. In sharp contrast, the poplar SVP-like (SVL), a MADS-box gene that regulates bud-break, is not regulated by histone marks^29^, while both *EBB1* and *EBB3* seem to be regulated by histone modifications^48^ (Fig. 3d). Thus, although the three genes appear to be in a common regulatory module, their larger regulatory context is different. *SVL* is subject to hormonal, while *EBB1* and *EBB3* to chromatin and hormonal regulation.

The discovery of the three genes and their interactions opens the door for a more comprehensive characterization of the genetic mechanisms regulating bud-break and enables approaches to modify dormancy-associated traits in shrubs and forest and fruit trees from temperate and boreal latitudes more rapidly and strongly. Such breeding and biotechnological approaches may become increasingly important in the face of rapid climate change, whose pace is likely to exceed the capacity of the forests to adapt through natural selection alone^49^. Poor synchronization of bud phenology with local climates can lead to significant damage from early and late frosts, poor crop yields, and the outbreak of pest and disease problems. A better understanding of the molecular mechanisms of the processes involved will allow for knowledge-based approaches to mitigate existing challenges.

## Methods

### Mutant *ebb3D* discovery

A poplar activation tagging population was generated and screened^34,50^. The *EARLY BUD-BREAK3D* (*ebb3D*) mutant was discovered in a field trial with 2-year-old plants in which all 4 ramets (arranged in 2 randomly distributed blocks with 2 ramets each) of the line flushed earlier than wild type. The early bud-break phenotype of the *ebb3D* mutant line was validated in a controlled growth chamber experiment, as described below.

### Molecular characterization of the mutant

The sequence flanking the insertion of the activation tag was recovered using TAIL-PCR^34,50^. The recovered sequence was positioned in the poplar genome by BLAST searches and proximal genes to the insertion site were studied for activation by comparing their expression in the *ebb3D* mutant and wild type plants (see below for details of the expression analysis).

### Plant material, growth conditions, and phenotypic measurements

Hybrid clones 717-IB4 (*Populus tremula x alba;* wildtype/WT-717) and T89 (*Populus tremula x tremuloides;* wildtype/WT-T89) were used in the experiments. The EBB1-OE line generated in the WT-717 background, and the SVL-OE and SVL-RNAi lines were generated in the WT-T89 background^29,34^. Plants were first cultivated in vitro in half-strength MS medium (Duchefa) under sterile conditions for 5 weeks and then transferred to small pots with soil for 2 weeks for acclimation to greenhouse conditions. Plants were then transferred to 2 L pots and grown for 4 weeks in the greenhouse (16-h light, 20 °C) before the manipulative dormancy experiments. For growth cessation and dormancy induction, plants were transferred from the greenhouse to growth chambers and grown for 10 weeks under short-day (SD) photoperiod (8-hour light, 20 °C /16-h dark, 18 °C). Height, numbers of leaves formed after initiation of SD treatment and bud-set at the apex were recorded weekly^34^ Dormant plants were then placed in a cold room at 4 °C for 5 weeks to meet the chilling requirement. To induce bud-break, plants were transferred to warm temperature and LD photoperiod (16-h light, 20 °C/8 h dark, 18 °C). Bud-break of the apical bud was monitored and scored daily^34^.

### Generation of plasmid constructs and transformation

*EBB3* full-length CDS was amplified using ebbD3attB1 forward 5′-GGGGACAAGTTTGTAC AAAAAAGCAGGCTATGGATGTGATGGTTTCAGCTC-3′ and ebb3DattB2 reverse 5′-GGG GACCACTTTGTACAAGAAAGCTGGGTTCAAAGCCCCTCGTCTTTGTAT-3′ primers and similarly, RPL34e using RPL34eattB1 forward 5′-GGGGACAAGTTTGTACAAAAAAGCAG GCTATGGTGCAGCGTCTGACTTAC-3′ and RPL34eattB2 reverse 5′-GGGGACCACTTTGTACAAGAAAGCTGGGTTCATTTTGAGGCCTGTTTTTCCT-3′ primers containing B1 and B2 recombination site sequences, respectively. The amplified fragments were first cloned into pDONR221 and then into destination vector pK2GW7 (CaMV 35S promoter) using Gateway BP and LR recombination reactions respectively (Invitrogen). The constructs were sequence-verified and transformed into *Agrobacterium* strain C58 using the freeze-thaw method^51^. To generate the EBB3-RNAi construct, a 200 bp fragment was amplified using full-length the *EBB3* cDNA as a template and the following primers (Forward 5′-CACCTCTGGC GGGACCGAAT-3′ and Reverse 5′-GCAGGCGGCATGACATGGCT-3′). The amplified fragment was cloned into pENTR/D-TOPO (Invitrogen), sequence verified, and then transferred into the plant transformation vectors (CaMV 35S promoter) to generate the EBB3-pK7GWIWG2 (I) construct, which was then transformed into *Agrobacterium* strain GV3101pmp90RK^52^. The EBB3-RNAi construct was transformed into WT-T89 and whereas EBB3-pK7GWIWG2 (I) construct was into WT-717 plants^53,54^. For expression analysis during the dormancy cycle, we collected vegetative apical buds from small branches of three individual wild aspen (*Populus tremuloides*, ~20-years old) trees for each month of the dormancy period (September to April). Whole buds were immediately frozen in liquid nitrogen. For tissue-specific expression analysis, different tissue samples were collected from greenhouse grown four months old WT-717 plants and stored at −80 °C until processed. In control condition experiments, shoot apices for gene expression analyses were collected at the same time of the day (i.e., 15:00 h), after 0, 10WSD, 2WC, 5WC, and 2WLD frozen in liquid nitrogen, and stored at −80 °C.

To generate GFP-tagged version of EBB1 and EBB3 DNA, we amplified EBB1 and EBB3 CDSs using gene specific primers (EBB1_B1F: 5′-GGGGACAAGTTTGTACAAAAAAGCAGGCTATGGAAGAAGCGCTTAGAAG-3′, EBB1_B2R: 5’-GGGGACCACTTTGTACAAGAAAGCTGGGTTAAAGCTGGCAGCAAAGGCACT-3′ and EBB3_B1F: 5′-GGGGACAAGTTTGTACAAAAAAGCAGGCTATGGATGTGATGGTTTCAGC-3′, EBB3_B2R: 5′-GGGGACCACTTTGTACAAGAAAGCTGGGTTAAGCCCCTCGTCTTTGTATT-3′) containing B1 and B2 recombination site sequences, respectively. The amplified fragments were cloned into destination vector pMDC83 (CaMV 35S promoter) using Gateway LR recombination reaction (Invitrogen). The sequence-verified plasmids were then used for the poplar protoplast transfection assays.

### Hormone treatments

For the ABA treatment, apices were cut and placed in MS solution with or without 50 µM ABA for 2 h and were then sampled for RNA extraction and expression analysis. For gibberellin (GA) treatments, 3 mM GA_3_ solution was applied directly to the shoot apex and sampled after 24 h for expression analysis^55^.

### RNA isolation and quantitative real-time PCR analysis

Total RNA was extracted using an RNeasy® Plant Mini Kit (Qiagen), or an Aurum Total RNA kit (Bio-Rad), or an RNeasy® Plus Universal Kit (Qiagen). Total RNA (10 µg) was treated with RNase-Free DNase (Qiagen) and cleaned using an RNeasy® Mini Kit (Qiagen). One µg of the RNA was used to generate cDNA using an iScript cDNA synthesis kit (Bio-Rad) or SuperScriptII (Invitrogen, Life Technologies). *ACT7* (WT-717 background) and *ubiquitin* (*UBQ*) (WT-T89 background) genes were selected and validated using geNorm Software^56^ as a reference gene. qRT-PCR analyses were carried out with StepOnePlus Real-Time PCR System (Applied Biosystems, Life Technologies) and Light Cycler 480 II (Roche) using Maxima SYBR Green qPCR Master Mix (Thermo Fisher Scientific Co.) and relative expression values were calculated using the ∆-Ct-method^12^. A complete list of the primers used for RT-PCR is presented in Supplementary Table 1.

### *Populus* protoplast isolation and DNA transfection

WT-717 poplar plants were grown in low light (60 µmol/m^2^) for 6–8 weeks and around 20 fully expanded leaves (1 g) were used to isolate protoplast^57,58^. Briefly, fully expanded leaves were cut into 0.5–1 mm fine strips and digested in 40 mL of enzyme solution (cellulase R10 3%, macerozyme R10 0.8%) in dark for 4–5 h without shaking and later 30 min shaking at 50 rpm to release the protoplasts. The protoplasts were harvested and diluted to a concentration of 4 × 10^7^ protoplast mL^−1^. The plasmids used in transfections were isolated using Qiagen Plasmid Midi Kit (Qiagen Inc.), and 40 µg of EBB1-GFP (EBB1-pMDC83) and 50 µg of EBB3-GFP (EBB3-pMDC83) plasmid DNA added to 500 µL (2 × 10^7^ protoplasts) of protoplast for each transfection assays.^58^ After transfection, protoplasts were incubated in dark for 20 h and then 4 h on ice. Successful expression of the GFP-tagged proteins was monitored by examining GFP fluorescence of the transfected poplar protoplasts using Olympus SZX16 (Center Valley, PA, USA) (Supplementary Fig. 7).

### Chromatin immunoprecipitation assays

After transfection and successful validation of GFP fluorescence, protoplasts were harvested by centrifugation at 200 × *g*, and the pellet washed with ice-cold 1× PBS. Formaldehyde (1% final concentration) was used to crosslink the protein to DNA, and 125 mM glycine was used to stop the reaction. The crosslinked protoplasts were used to isolate the chromatin using EpiQuick ChIP Kit (EPIGENTEK, P-2002). Anti-GFP (Abcam, Cat No. ab290) and IgG (negative control, provided with kit) antibodies were used for chromatin immunoprecipitation. The enrichment of bound target sequences was quantified by qRT-PCR using the DNA recovered from anti-GFP and IgG ChIPs and normalized against 5% input DNA. All ChIP experiments were carried out in three biological replicates.

### Generation of labeled *SVL* promoter fragments and EMSAs with EBB1 and EBB3 proteins

Sequence analysis of the *SVL* promoter showed the presence of an ERF binding site (GCC-box, GCCGCCA) ~150 bp upstream (−150 bp) of the translation start site. Two sets of primers that can amplify 150 bp fragments, one with the binding site and the other without the binding site were amplified by using biotin-labeled primers (see Supplementary Table 1) and *P. trichocarpa* genomic DNA. The fragments were purified using an E.Z.N.A. Gel Purification Kit (Omega Bio-tek) prior to use in gel-shift assays. EMSAs were performed using biotin-labeled promoter fragments and cell extracts from *Arabidopsis* protoplasts expressing HA-EBB1/HA-EBB3 or control extracts from non-transformed protoplasts^11^. After confirming that HA-EBB1/HA-EBB3 bound to the *SVL* promoter fragment, smaller, biotin-labeled F1 fragments (27 bp) containing the GCC-box (GCCGCC) or a mutated version of the GCC-box were synthesized. For the EMSA reaction, 10 μL protoplast cell extract was mixed with 0.5 μL biotin-labeled DNA (10 fmol/mL), 0.4 μL nonspecific competitor (poly [dI:dC], 1 mg/mL), and 0.5 μL BSA (20 mg/mL) in EMSA buffer. To determine binding specificity, mutated GCC-box fragments were also used. The mixtures were incubated on ice for 10 min followed by 30 min at room temperature to allow binding. The samples were then separated on a non-denaturing polyacrylamide gel (5%) prepared with 0.5XTBE and transferred to a Hybond N + membrane (GE Healthcare). Finally, a Light Shift Chemiluminescent EMSA kit (Pierce) was used for cross-linking and detection.

### Generation of *CYCD3.1* GCC-box probe and EMSAs with EBB3 protein

The CDS of *PtaEBB3* was amplified using forward 5′-AAAAGAATTCATGGATGT GATGGTTT-3′ and reverse 5′-AAAAGCGGCCGCAAGCCCCTCGTCTTTG-3′ primers and cloned into the pET32a(+) vector at the *EcoRI*/*NotI* sites. The construct was sequenced validated and transformed into One Shot™ BL21(DE3) *E. coli* cells (Invitrogen). Colonies growing on selection media were validated using PCR and restriction digests. Successfully validated colonies were used to inoculate 50 mL LB medium grown for 2 h at 37 °C to obtain an OD_600_ between 0.6 and 0.8 and then induced for 20 h with 0.1 mM IPTG (Isopropyl β-d-thiogalactoside) at 16 °C. The PtaEBB3-His protein was purified on Ni^2+^‐NTA agarose resin (Qiagen) following the manufacturer’s protocol. Forward and reverse primers containing the ERF binding site (GCC-box, GCCGGGCCA) were synthesized (Eurofins Genomics). The double‐stranded probe was prepared by annealing the forward and reverse primers. The EMSA assay was carried out using the Electrophoretic Mobility-Shift Assay (EMSA) Kit, with SYBR™ Green and SYPRO™ Ruby EMSA stains (Invitrogen), according to the product instructions. Protein-DNA complex samples were loaded onto a 4–20% gradient polyacrylamide native gel (Bio-Rad) and run at 100 V for 1 h. The amount of DNA and protein used in the various experiments are indicated in Fig. 6. The nucleic acid in the gel was stained using SYBR® Green and visualized using a Gel Doc-It (UVP). The primers used for the EMSA analysis are listed in Supplementary Table 1.

### ChIP-seq experiment

To perform ChIP-seq experiment, apical buds from WT plants of three biological replicates were collected from before dormancy inductive treatments (0 W), after 6 and 10 weeks under SD conditions (6WSD and 10WSD), and again after an additional 4 weeks of cold treatment (4WC). Anti-trimethyl-Histone H3 (LysK27) (Millipore, Cat No. #07-449) and anti-Histone H3 (Abcam, Cat No. ab1791) antibodies were used for chromatin immunoprecipitation. Paired-end (PE) sequencing was done by BGI-Tech. Sequencing reads were processed following the guidelines described at http://www.epigenesys.eu/en/protocols/bio-informatics/1283-guidelines-for-rna-seq-data-analysis. Reads quality was assessed using FastQC (http://www.bioinformatics.babraham.ac.uk/projects/fastqc/), v0.11.4. Ribosomal RNA (rRNA) reads were quantified and filtered using SortMeRNA^59^. Reads were filtered to remove adapters and trimmed for quality using Trimmomatic and then mapped to the *Populus* genome using STAR^60,61^. Then reads were remapped using BWA-MEM^62^ and peaks were called genome-wide using MACS2^63^ with the non-default parameters^29^, on sequencing libraries down-sampled to 10 million PE reads. The depth of this down-sampled library was estimated by an ad hoc saturation/rarefaction analysis based on the number of peaks identified by MACS2 in varying subsets of the original dataset. These downstream analyses were solely used to estimate the fraction of the genome mapped under the different growing conditions. The data were normalized using ratios obtained for the analysis of the *EBB3* locus histone methylation status.

Reads mapped to the sequence of the *EBB3* gene including 2 kb upstream and downstream region were extracted from the alignment. Coverage was calculated in the above region, transformed to log2, and corrected for the abundance differences between samples (i.e., the fraction of the genome mapped under the different growing conditions in the 10 M PE read subset; the latter selection addressing any library size factor scaling otherwise required). Finally, the H3K27me3 abundance was normalized by H3 abundance, the mean of three biological replicates was calculated and curves were fitted to the data by local polynomial regression. R^64^ and Bioconductor^65^ were used to compare differences in histone methylation between the time points.

### RNA sequencing and differential expression analyses

Illumina HiSeq 2500 was used to perform RNA-seq for 125 cycles in paired-end mode at BGI (Beijing, China) using RNA isolated from apices of wild type and EBB3-RNAi at 10 weeks SD, 2 W, and 5 W cold and 2 W LDs. Briefly, the quality of the raw sequence data was assessed using FastQC (http://www.bioinformatics.babraham.ac.uk/projects/fastqc/). Residual ribosomal RNA (rRNA) contamination was assessed and filtered using SortMeRNA^59^ (settings --log --paired_in --fastx--sam --num_alignments 1) using the rRNA sequences provided with SortMeRNA (rfam-5s-database-id98.fasta, rfam-5.8s-database-id98.fasta, silva-arc-16s-database-id95.fasta, silva-bac-16s-database-id85.fasta, silva-euk-18s-database-id95.fasta, silva-arc-23s-database-id98.fasta, silva-bac-23s-database-id98.fasta and silva-euk-28s-database-id98.fasta). Data were then filtered to remove adapters and trimmed for quality using Trimmomatic^60^ (v0.36; settings TruSeq3-PE-2.fa:2:30:10 LEADING:3 SLIDINGWINDOW:5:20 MINLEN:50). After both filtering steps, FastQC was run again to ensure that no technical artefacts were introduced. Filtered reads were pseudo-aligned to v1.1 of the *P. tremula* transcripts^66^ using kallisto^67^ (v0.44; non default settings: -b 100 --rf-stranded -t 8). Statistical analysis of single-gene differential expression between conditions was performed in R^68^ (v3.6.0) using the Bioconductor^69^ (v3.9) DESeq2 package^70^ (v1.20.1). FDR (False Discovery Rate) adjusted *P*-values were used to assess significance; a common threshold of 1% was used throughout. For the data quality assessment (QA) and visualisation, the read counts were normalised using a variance stabilising transformation as implemented in DESeq2.

### Reporting summary

Further information on research design is available in the Nature Research Reporting Summary linked to this article.

## Supplementary information

Supplementary information

Peer Review

Reporting Summary

Description of Additional Supplementary Files

Supplementary Data 1-9

## Data Availability

Data supporting the findings of this work are available within the paper and its Supplementary Information files. A reporting summary for this Article is available as a Supplementary Information file. The datasets and plant materials generated and analyzed during the current study are available from the corresponding author upon request. The ChIP-seq data were deposited at the European Nucleotide Archive under the accession number PRJEB42484. The raw RNA-seq data are available from the European Nucleotide Archive under the accession number PRJEB35768. An overview of the RNA-seq data, including raw and post-QC read counts and alignment rates is available from GitHub [https://github.com/nicolasDelhomme/poplar-early-bud-break]. Source data are provided with this paper.

## Source data

Source Data

## Code availability

The custom R scripts used to assess the biological relevance of the data using principal component analysis (PCA) and other visualisations (e.g., heatmaps) is available at GitHub [https://github.com/nicolasDelhomme/poplar-early-bud-break]. The R script used to reproduce the *EBB3* gene locus analysis is available in Zenodo [https://doi.org/10.5281/zenodo.4395779].
