# Supplementary material for: EARLY BUD-BREAK 1 and EARLY BUD-BREAK 3 control resumption of poplar growth after winter dormancy
Source: Nat Commun. 2021 Feb 18;12:1123. doi: 10.1038/s41467-021-21449-0 (PMC7893051; doi:10.1038/s41467-021-21449-0)
Supplement: Supplementary file 1 — Supplementary information [file 41467_2021_21449_MOESM1_ESM.pdf]

# EARLY BUD-BREAK 1 and EARLY BUD-BREAK 3 control resumption of poplar growth after winter dormancy

Azeez *et al*.

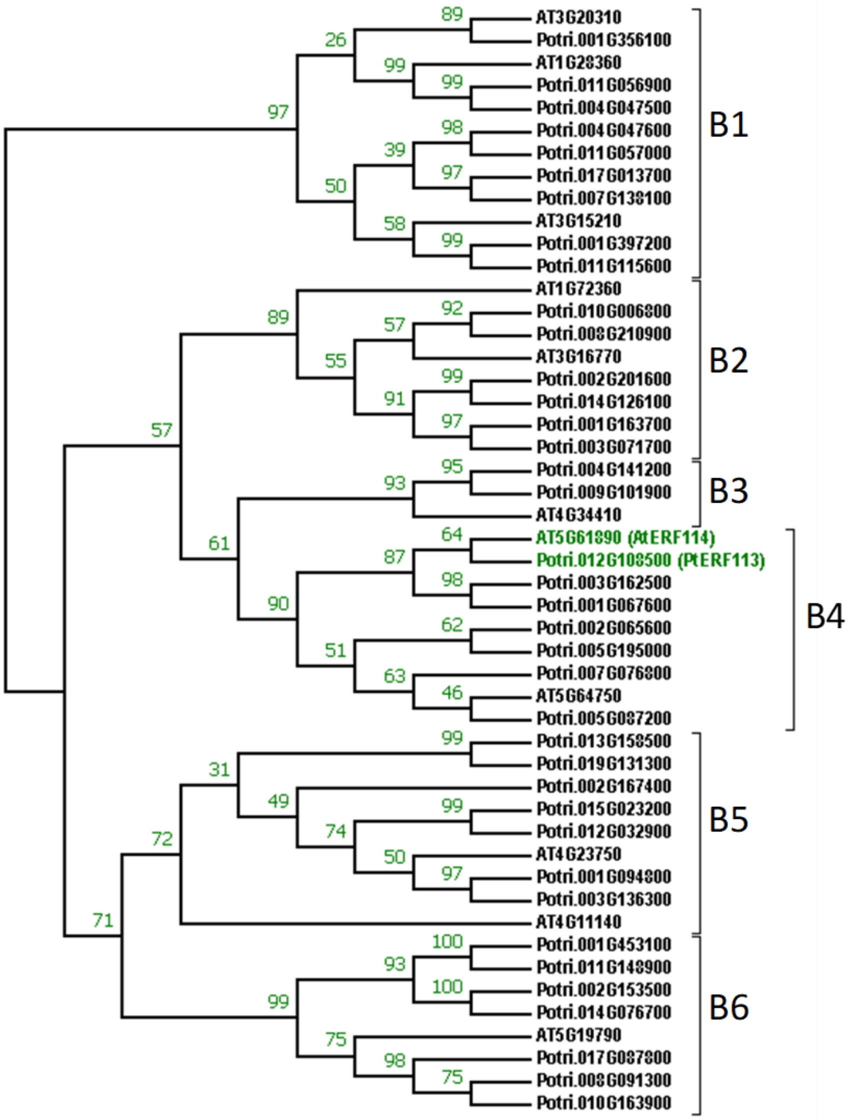


**Supplementary Figure 1. Phylogenetic analysis of EBB3 proteins in *Populus* and *Arabidopsis*.** Phylogenetic analysis of *Populus* and *Arabidopsis* AP2/ERF proteins of subfamilies B1, B2, B3, B4, B5, and B6. *Populus* proteins begin with Potri, and *Arabidopsis* proteins begin with AT. The phylogenetic tree was constructed using MEGA7. Numbers at the tree branches indicate percent bootstrap support of 1000 iterations. EBB3 and the close *Arabidopsis* ortholog are indicated in green. Source data are provided as a Source Data file.

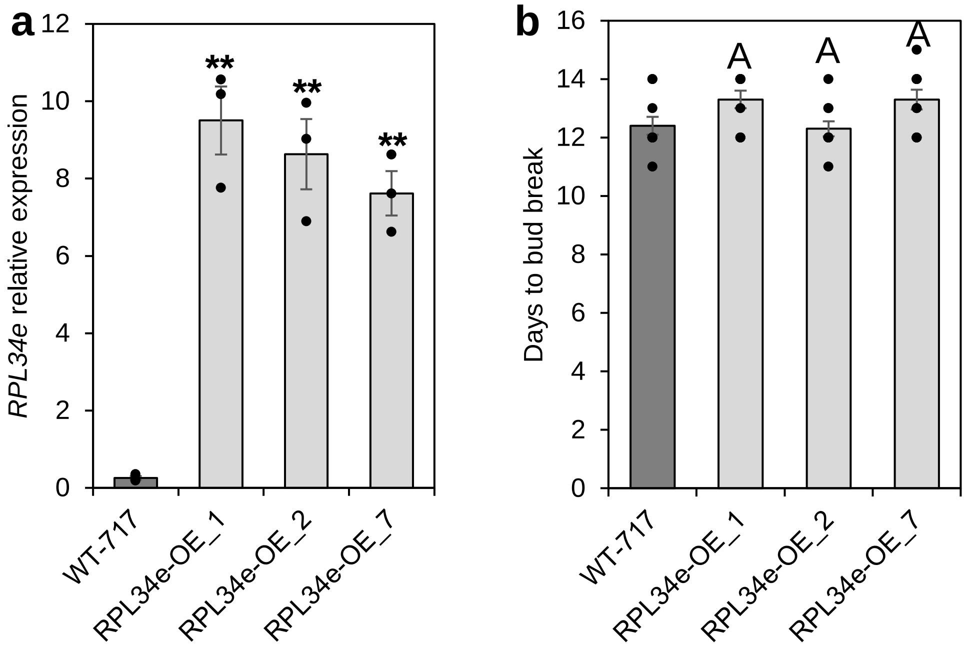


**Supplementary Figure 2. *RPL34e* overexpressing lines show no change in bud-break compared to WT.** (a) Relative expression of *RPL34e/*Potri.012G108400 in RPL34e-OE lines normalized to *ACT7* gene. Expression values are the average of three biological replicates ± SEM (b) Time to bud-break relative to WT-717 control plants in RPL34e-OE lines. Values are the average of three biological replicates ± SEM. Asterisks (**) indicate highly significant differences at $P<0.001$, whereas letters (A) on top indicate no significant differences compared to their respective control ($P>0.8$) and determined by two-tailed paired *t*-tests. Source data underlying Supplementary Figure 2b are provided as a Source Data file.

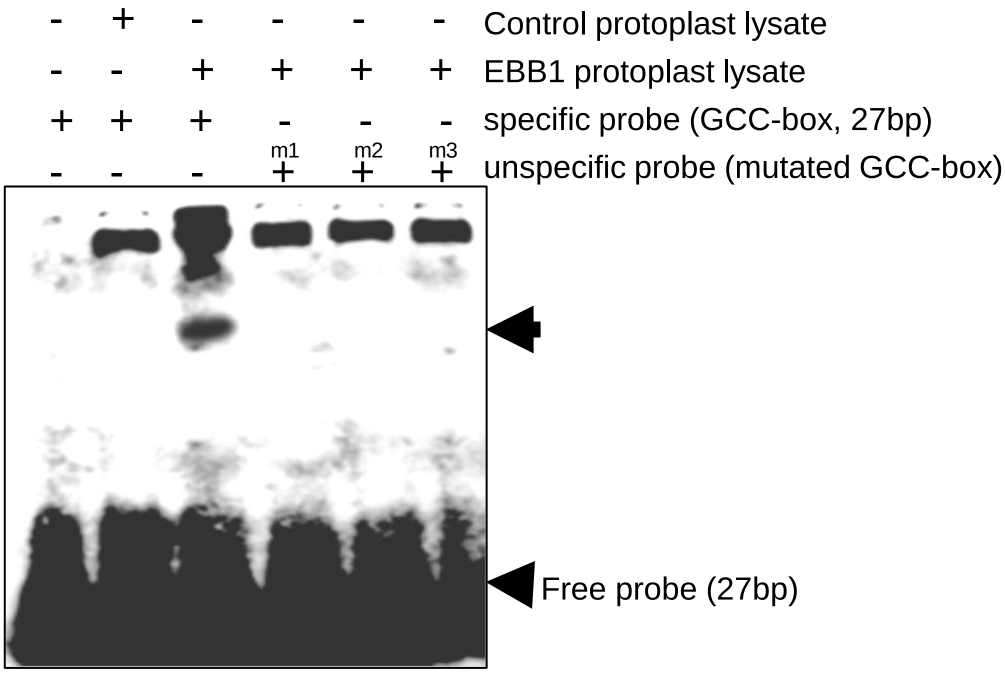


**Supplementary Figure 3. EBB1 binds to the *SVL* promoter.** HA-tagged EBB1 protein bind to a GCC-box in the *SVL* promoter. Different components used in EMSA reaction mixtures are as indicated on top. Source data are provided as a Source Data file.

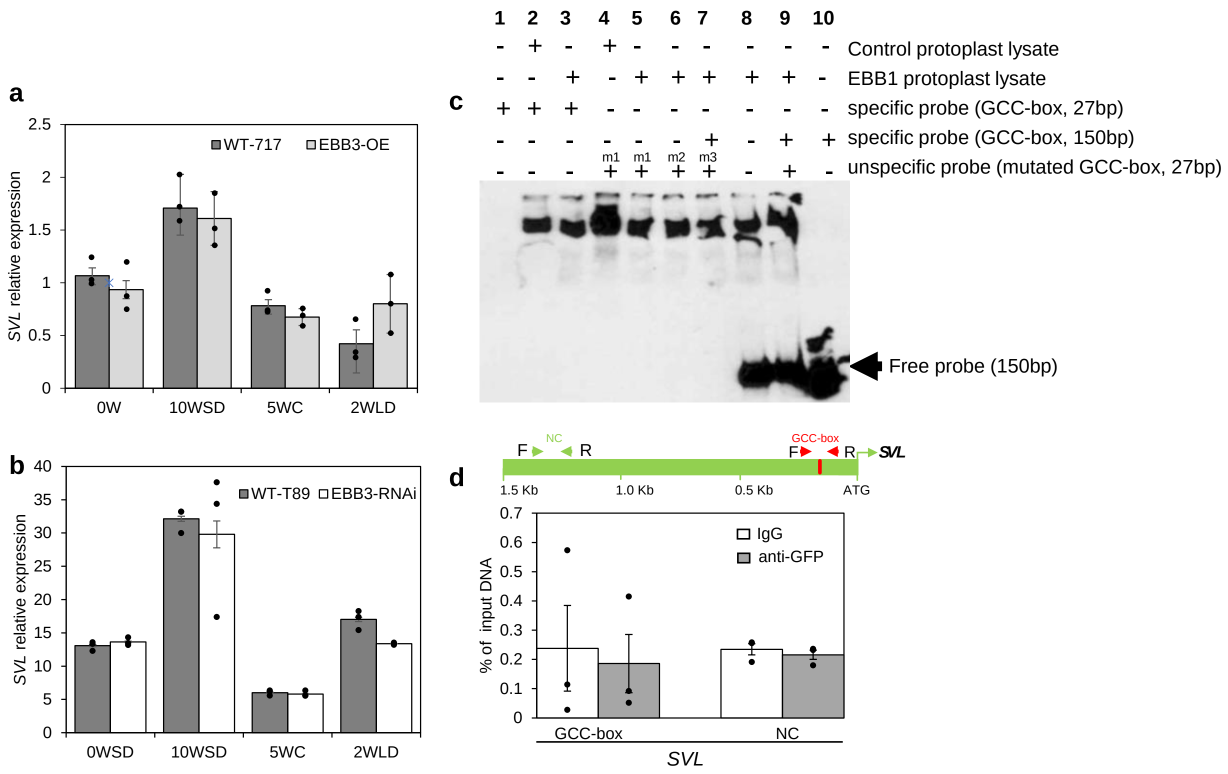


**Supplementary Figure 4. EBB3 does not bind to the *SVL* promoter.** Relative expression of *SVL* in EBB3 over-expressing (a) and under-expressing (b) plants, Expression values are the average of three biological replicates ± SEM. *UBQ* and *ACT7* were used to normalize *SVL* expression in T89 and 717 backgrounds respectively. Expression values are the average of three biological replicates ± SEM. No significant differences compared to their respective control, determined by using *t*-test. (c) HA-tagged EBB3 protein does not bind to the *SVL* promoter. Components used in EMSA reaction mixtures are as follows, 1- Free probe, 2- F1 (27bp) with control (no protein) extract, 3- EBB3 protein extract with F1 fragment, 4-7- F1 Mutated fragments with EBB3 protein, 8- 150bp *SVL* promoter fragment containing GCCGCC box with EBB3 protein, 9- *SVL* promoter fragment without GCCGCC box with EBB3 protein, 10- Free probe (no protein) of the 150bp *SVL* promoter fragment (d) Enrichment of a DNA fragment in the *SVL* promoter containing a GCC-box and quantified by ChIP-qPCR. The green box is a schematic representation of the *SVL* promoter showing the position of the GCC-box (red). Red arrows delineate the position of DNA fragment containing the GCC-box and green arrows demarcate the position of DNA fragments with no GCC-box used as negative control (NC) in ChIP-qPCR analysis. Chromatin from EBB3-GFP DNA transfected poplar protoplasts was isolated using anti-GFP antibody and IgG used as a control antibody. ChIP-purified DNA was used to perform ChIP-qPCR, values are represented as the percentage of input (% of input) DNA. Values are the average of three biological replicates ± SEM. No significant differences compared to their respective control ($P$>0.8), determined by two-tailed paired multiple *t*-tests. Source data underlying Supplementary Figure 4c and 4 d are provided as a Source Data file.

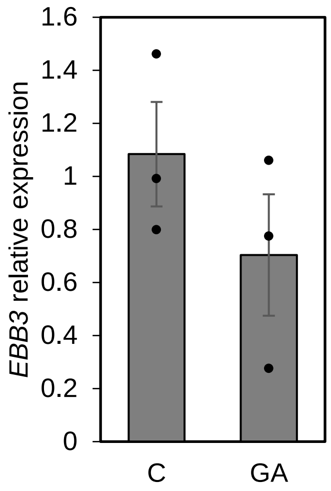


**Supplementary Figure 5. GA does not affect *EBB3* expression.** Relative expression of *EBB3* in control (C, untreated) and GA-treated WT-717 apices. Expression values are the average of three biological replicates ± SEM, *ACT7* was used to normalize expression. No statistically significant differences between control and GA treatments were found at $P<0.05$ as determined by two-tailed paired *t*-tests. See Methods for details of the GA treatment.

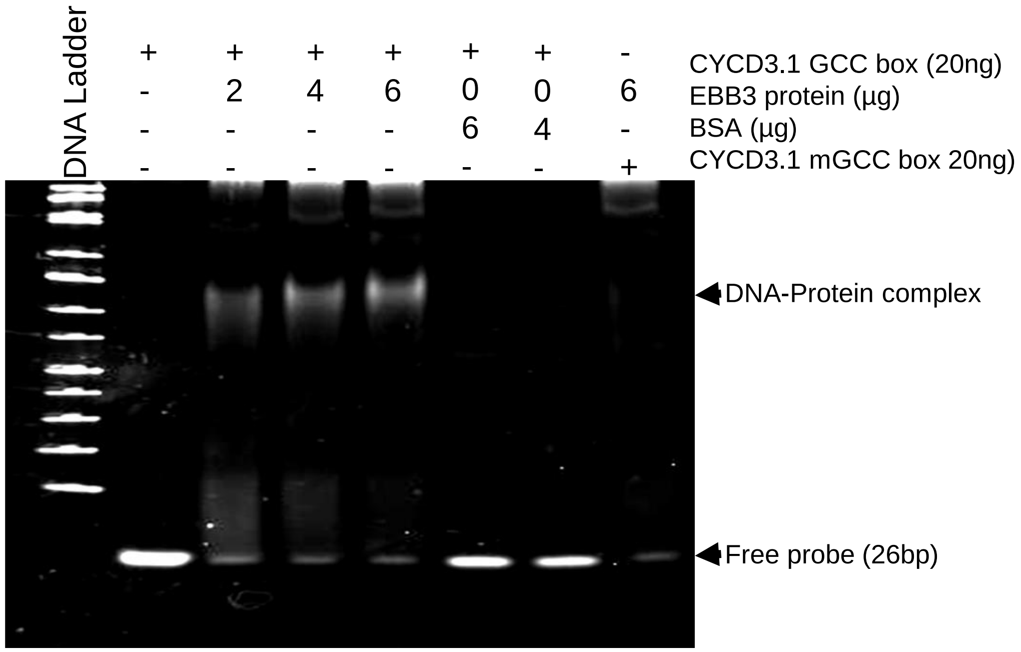


**Supplementary Figure 6. EBB3 binds to the *CYCD3.1* promoter in vitro in EMSA assay.** 6xHIS-EBB3 protein binds to GCC-box in the *CYCD3.1* promoter. Different components used in the electrophoretic mobility shift (EMSA) reaction mixtures are as indicated on the image. The specificity was demonstrated by varying the concentration of EBB3 protein with 20ng of a probe (GCC-box, GCCGGGCCA). BSA was used as a negative control. The EBB3 protein did not bind when a mutated GCC-box (mGCC-box, TCCTTTCCA) was used. Source data are provided as a Source Data file.

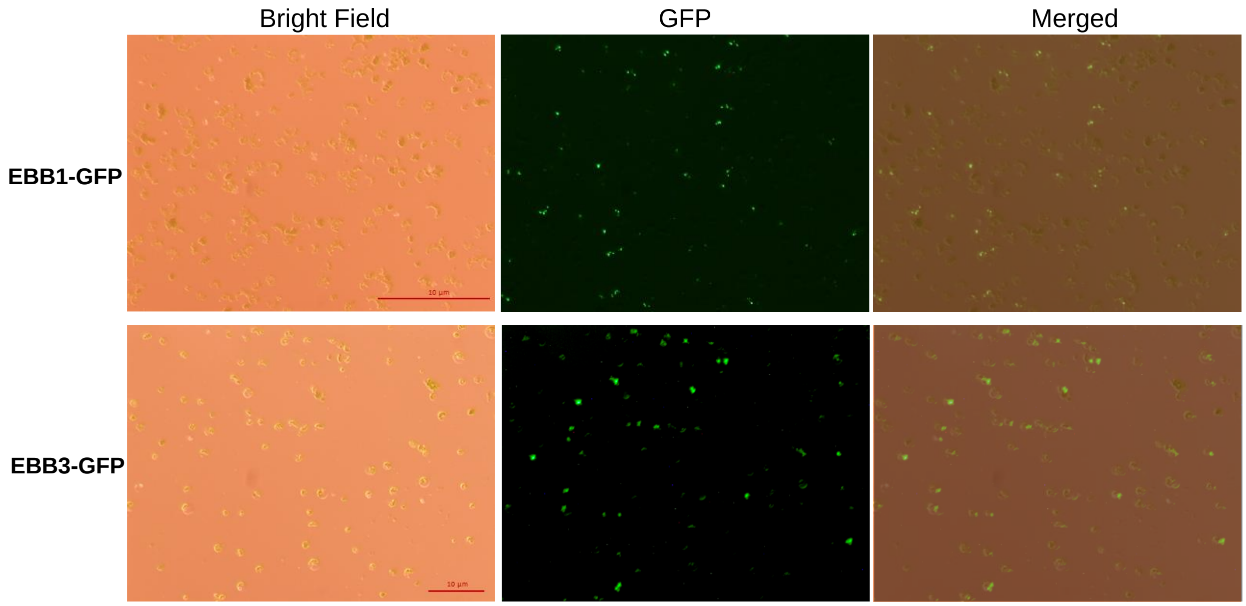


**Supplementary Figure 7. Poplar protoplasts expressing the EBB1-GFP (top) and EBB3-GFP (bottom) fusion proteins after transfection.** The same protoplasts were used for the ChIP-qPCR experiments as described in the text. Successful expression of the tagged proteins was confirmed by GFP visualization (Scale bar, 10 µm). Source data are provided as a Source Data file.

**Supplementary Table 1. List of primers used in this study.**

| Used in 717 background | | |
|---|---|---|
| Primer name | Sequence (5'-3’) | Purpose |
| PtaGA20ox1_F | TTCCACAACGAGAGCGGTCTTG | RT-PCR |
| PtaGA20ox1_R | TTTGAGAGGCAGGGAAGGGAGAG | RT-PCR |
| PtaGA20oxi2_F | GCAGTTCATTTGGCCAGACA | RT-PCR |
| PtaGA20oxi2_R | CACAGGACTCGCCAATCTTT | RT-PCR |
| PtaCYCD3.1_F | CAAGTGCCCCTTTTATTGGA | RT-PCR |
| PtaCYCD3.1_R | TAGGCCGAGCCTTCTAGTGA | RT-PCR |
| PtaFT1-rt_F | CAACTGGGGCAAGCTTTGGCCATGAAAC | RT-PCR |
| PtaFT1-rt_R | TTATCGCCTCCTACCACCAGAGCCAC | RT-PCR |
| PtaSVL-rtF | TGAGAGACTCAAACAGCAAGTG | RT-PCR |
| PtaSVL-rtR | ACTGCCCTTCCTCGTAACCAAC | RT-PCR |
| PtaAct7-F | TGGCCGATGCCGAGGATATTCAAC | RT-PCR |
| PtaAct7-R | ATCACCTGCAAACCCAGCCTTCAC | RT-PCR |
| PtaEBB3-rtF | AAGGGAATGCAAGGAGACGA | RT-PCR |
| PtaEBB3-rtR | GCCAGATGGAACCCTTTCAG | RT-PCR |
| CYCD3.1-emsa-F1 | TGAAAGCTGGCCGGGCCAAGTCTCCA | EMSA |
| CYCD3.1-emsa-R1 | TGGAGACTTGGCCCGGCCAGCTTTCA | EMSA |
| CYCD3.1-emsa-mF1 | TGAAAGCTGTCCTTTCCAAGTCTCCA | EMSA |
| CYCD3.1-emsa-mR1 | TGGAGACTTGGAAAGGACAGCTTTCA | EMSA |
| SVL-ChIP_F | ACTAGGGTTTGTCACAGGTTCCTCTC | ChIP-qPCR |
| SVL-ChIP_R | CAAAGAGAAGAACTGGGTTAGTGCTAC | ChIP-qPCR |
| SVL-ChIP-NC_F | CTTACAGGAGAGGCAGGAACAAGAAC | ChIP-qPCR |
| SVL-ChIP-NC_R | TGGGACCGGCTTAAAAATATTGTATC | ChIP-qPCR |
| CYCD3.1-ChIP_F | GAACCGAGCTACAAGTAGACGAC | ChIP-qPCR |
| CYCD3.1-ChIP_R | TCTGAGCTGGTATGATAGGTGGAC | ChIP-qPCR |
| CYCD3.1-ChIP-NC_F | CGACACTTAGAGCTTGGCACTTG | ChIP-qPCR |
| CYCD3.1-ChIP-NC_R | TCCATGCATACGAGGGAATGAC | ChIP-qPCR |
| Used in T89 background | | |
| Primer name | Sequence (5'-3’) | Purpose |
| PttEBB3-F | AAGGGAATGCAAGGAGACGA | RT-PCR |
| PttEBB3-R | GCCAGATGGAACCCTTTCAG | RT-PCR |
| PttEBB1-F | GGGAACATGAGGTACCGTGG | RT-PCR |
| PttEBB1-R | CCAACGCCGTTCCTTAGACT | RT-PCR |
| PttSVL-F | TGAGAGACTCAAACAGCAAGTGG | RT-PCR |
| PttSVL-R | ACTGCCCTTCCTCGTAACCAAC | RT-PCR |
| PttFT1-F | CAACTGGGGCAAGCTTTGGCCATGAAAC | RT-PCR |

| | | |
|---|---|---|
| PttFT1-R | TTATCGCCTCCTACCACCAGAGCCAC | RT-PCR |
| PttGa20-Oxidase1-F | TTCCACAACGAGAGCGGTCTTG | RT-PCR |
| PttGa20-Oxidase1-R | TTTGAGAGGCAGGGAAGGGAGAG | RT-PCR |
| PttGa20-Oxidase2-F | GCAGTTCATTTGGCCAGACA | RT-PCR |
| PttGa20-Oxidase2-R | CACAGGACTCGCCAATCTTT | RT-PCR |
| SVPprom F1 (150bp with GCC-box) | GAATTCTCTATCTAGGGTTTGTCAC | EMSA |
| SVPprom R1 (150bp with GCC-box) | GTCGATCTTTTTTATCTGAATCC | EMSA |
| SVPprom F2 (150bp without GCC-box) | CGATATCTCGCGGTACTTTACTATCGC | EMSA |
| SVPprom R2 (150bp without GCC-box) | GCTAAATTTAGAGAGGAACCTGTGAC | EMSA |
| SVL Prom F1 (27bp, GCC-box) | TTAGCAAAATGCCGCCTGTTTTACTCC | EMSA |
| SVL Prom R1 (GCC-box) | GGAGTAAAACAGGCGGCATTTTGCTAA | EMSA |
| SVL Prom F1m1 | TTAGCAAAATGCAGCATGTTTTACTCC | EMSA |
| SVL Prom R1m1 | GGAGTAAAACATGCTGCATTTTGCTAA | EMSA |
| SVL Prom F1m2 | TTAGCAAAATGACGACTGTTTTACTCC | EMSA |
| SVL Prom R1m2 | GGAGTAAAACAGTCGTCATTTTGCTAA | EMSA |
| SVL Prom F1m3 | TTAGCAAAATTTCTTCTGTTTTACTCC | EMSA |
| SVL Prom R1m3 | GGAGTAAAACAGAAGAAATTTTGCTAA | EMSA |

**Supplementary Table 2. Numbers of statistically significant (*P*<0.05 FDR) transcriptomic changes during dormancy associated with with EBB3 gene suppression.**

| Time point | Upregulated | Downregulated |
|---|---|---|
| 10WSD | 10 | 19 |
| 2WC | 0 | 5 |
| 5WC | 79 | 31 |
| 2WLD | 750 | 412 |

Note: FDR: false discovery rate; W: weeks; SD: short days; LD: long days; C: cold (see details in Methods).
