# Supplementary material for: EARLY BUD-BREAK 1 and EARLY BUD-BREAK 3 control resumption of poplar growth after winter dormancy
Source: Nat Commun. 2021 Feb 18;12:1123. doi: 10.1038/s41467-021-21449-0 (PMC7893051; doi:10.1038/s41467-021-21449-0)
Supplement: Supplementary file 3 — Reporting Summary [file 41467_2021_21449_MOESM3_ESM.pdf]

nature research

Corresponding author(s): NCOMMS-19-40316C

Last updated by author(s): Jan 14, 2021

Reporting Summary

Nature Research wishes to improve the reproducibility of the work that we publish. This form provides structure for consistency and transparency in reporting. For further information on Nature Research policies, see our Editorial Policies and the Editorial Policy Checklist.

Statistics

For all statistical analyses, confirm that the following items are present in the figure legend, table legend, main text, or Methods section.

n/a	Confirmed	
☐	☒	The exact sample size (n) for each experimental group/condition, given as a discrete number and unit of measurement
☒	☐	A statement on whether measurements were taken from distinct samples or whether the same sample was measured repeatedly
☐	☒	The statistical test(s) used AND whether they are one- or two-sided<br>*Only common tests should be described solely by name; describe more complex techniques in the Methods section.*
☒	☐	A description of all covariates tested
☐	☒	A description of any assumptions or corrections, such as tests of normality and adjustment for multiple comparisons
☐	☒	A full description of the statistical parameters including central tendency (e.g. means) or other basic estimates (e.g. regression coefficient) AND variation (e.g. standard deviation) or associated estimates of uncertainty (e.g. confidence intervals)
☐	☒	For null hypothesis testing, the test statistic (e.g. F, t, r) with confidence intervals, effect sizes, degrees of freedom and P value noted<br>*Give P values as exact values whenever suitable.*
☒	☐	For Bayesian analysis, information on the choice of priors and Markov chain Monte Carlo settings
☒	☐	For hierarchical and complex designs, identification of the appropriate level for tests and full reporting of outcomes
☒	☐	Estimates of effect sizes (e.g. Cohen's d, Pearson's r), indicating how they were calculated

Our web collection on statistics for biologists contains articles on many of the points above.

Software and code

Policy information about availability of computer code

Data collection	https://doi.org/10.5281/zenodo.4395779
Data analysis	http://www.bioinformatics.babraham.ac.uk/projects/fastqc/<br>https://ebi.ac.uk/ena

For manuscripts utilizing custom algorithms or software that are central to the research but not yet described in published literature, software must be made available to editors and reviewers. We strongly encourage code deposition in a community repository (e.g. GitHub). See the Nature Research guidelines for submitting code & software for further information.

Data

Policy information about availability of data

All manuscripts must include a data availability statement. This statement should provide the following information, where applicable:

- Accession codes, unique identifiers, or web links for publicly available datasets
- A list of figures that have associated raw data
- A description of any restrictions on data availability

The authors declare that all data supporting the findings of this study are included in the main manuscript file, in the supplementary information or are available from the corresponding author upon request. The data necessary to reproduce the EBB3 gene locus analysis is available from the UPSCb GitHub repository (https://github.com/nicolasDelhomme/poplar-early-bud-break) in the form of bam files. The bam files are subset to the EBB3 +2kb upstream/downstream locus only.

Field-specific reporting

Please select the one below that is the best fit for your research. If you are not sure, read the appropriate sections before making your selection.

☐ Life sciences ☐ Behavioural & social sciences ☒ Ecological, evolutionary & environmental sciences

For a reference copy of the document with all sections, see nature.com/documents/nr-reporting-summary-flat.pdf

Ecological, evolutionary & environmental sciences study design

All studies must disclose on these points even when the disclosure is negative.

Study description

Activation Tagging, Field Trial, and Screening for Bud Phenology. The activation tagging population was generated in the 717-1B4 (Populus tremula × Populus alba) genotype as referred to as WT-717. A field trial of 627 independent transformation events and untransformed WT-717 plants was established in western Oregon. Each event was represented by four ramets. Measuring the time of bud-break was performed by daily visits and scoring bud-break. Events that displayed changes in their bud phenology in all four ramets were clonally propagated, and the observed phenotype was validated in a controlled growth chamber experiment.

Growth cessation and dormancy Induction. For growth cessation and dormancy induction, plants were transferred from the greenhouse to growth chambers and grown for 10 weeks under short day (SD) photoperiod (8-hour light, 20°C /16-hour dark, 18°C). Numbers of leaves formed after initiation of SD treatment and bud-set at the apex were recorded weekly. Dormant plants were then placed in a cold room at 4oC for 5 weeks to meet the chilling requirement. To induce bud-break, plants were transferred to warm temperature and long day (LD) photoperiod (16-hour light, 20°C /8 hour dark, 18°C). Bud-break was scored when bud swelling and emergence of green leaves were observed.

Research sample

Poplar's shoot apex

Sampling strategy

Samples for expression analysis and CHIP-seq: Apex samples were taken just before transferred under SDs (0W), and after plants had ceased growth and developed dormancy, i.e. after 10 weeks of short days (10WSD) and after; both 2 weeks (2WC) and 5 weeks (5WC) of exposure to low temperature (4 °C) to induce dormancy release; and followed by two weeks after the transfer to long day/ warm temperature conditions (2WLD). Three biological replicates used for each experiment and pool of six apices used for each time point.

Data collection

To score the number of leaves after SDs exposure and bud-break after cold treatment at least 10 plants were used for each genotype. Pictures of apices were taken using a Canon EOS digital camera.

Timing and spatial scale

Indicate the start and stop dates of data collection, noting the frequency and periodicity of sampling and providing a rationale for these choices. If there is a gap between collection periods, state the dates for each sample cohort. Specify the spatial scale from which the data are taken

Data exclusions

No data was excluded from analyses

Reproducibility

Each experiment included in the manuscripts repeated thrice

Randomization

Describe how samples/organisms/participants were allocated into groups. If allocation was not random, describe how covariates were controlled. If this is not relevant to your study, explain why.

Blinding

Describe the extent of blinding used during data acquisition and analysis. If blinding was not possible, describe why OR explain why blinding was not relevant to your study.

Did the study involve field work? ☒ Yes ☐ No

Field work, collection and transport

Field conditions

Describe the study conditions for field work, providing relevant parameters (e.g. temperature, rainfall).

Location

State the location of the sampling or experiment, providing relevant parameters (e.g. latitude and longitude, elevation, water depth).

Access & import/export

Describe the efforts you have made to access habitats and to collect and import/export your samples in a responsible manner and in compliance with local, national and international laws, noting any permits that were obtained (give the name of the issuing authority, the date of issue, and any identifying information).

Disturbance

Describe any disturbance caused by the study and how it was minimized.

Reporting for specific materials, systems and methods

We require information from authors about some types of materials, experimental systems and methods used in many studies. Here, indicate whether each material, system or method listed is relevant to your study. If you are not sure if a list item applies to your research, read the appropriate section before selecting a response.

Materials & experimental systems

n/a	Involved in the study
☐	☒ Antibodies
☒	☐ Eukaryotic cell lines
☒	☐ Palaeontology and archaeology
☒	☐ Animals and other organisms
☒	☐ Human research participants
☒	☐ Clinical data
☒	☐ Dual use research of concern

Methods

n/a	Involved in the study
☐	☒ ChIP-seq
☒	☐ Flow cytometry
☒	☐ MRI-based neuroimaging

Antibodies

Antibodies used: Anti-trimethyl-Histone H3 (LysK27) (Millipore, Cat No. #07-449), anti-Histone H3 (Abcam, Cat No. ab1791) and anti-GFP (Abcam, Cat No. ab290) antibodies were used for chromatin immunoprecipitation

Validation: These antibodies were validated as instructed by the manufacturers

ChIP-seq

Data deposition

☒ Confirm that both raw and final processed data have been deposited in a public database such as GEO.

☒ Confirm that you have deposited or provided access to graph files (e.g. BED files) for the called peaks.

Data access links
May remain private before publication.

https://doi.org/10.5281/zenodo.4395779
https://www.ebi.ac.uk/ena

Files in database submission

1. ChIP-Seq data for EBB3/data/EBB3
2. ChIP-Seq data for EBB3/doc
3. ChIP-Seq data for EBB3/pipeline
4. ChIP-Seq data for EBB3/report
5. ChIP-Seq data for EBB3/src/R
6. .gitignore
7. LICENSE
8. README.md

RNA-Seq data

Genome browser session
(e.g. UCSC)

https://doi.org/10.5281/zenodo.4395779
https://www.ebi.ac.uk/ena

Methodology

Replicates: Three biological replicates

Sequencing depth: *Describe the sequencing depth for each experiment, providing the total number of reads, uniquely mapped reads, length of reads and whether they were paired- or single-end.*

Antibodies: Anti-trimethyl-Histone H3 (LysK27) (Millipore, Cat No. #07-449) and anti-Histone H3 (Abcam, Cat No. ab1791)

Peak calling parameters: *Specify the command line program and parameters used for read mapping and peak calling, including the ChIP, control and index files used.*

Data quality: *Describe the methods used to ensure data quality in full detail, including how many peaks are at FDR 5% and above 5-fold enrichment.*

Software: https://doi.org/10.5281/zenodo.4395779
