# Supplementary material for: EARLY BUD-BREAK 1 and EARLY BUD-BREAK 3 control resumption of poplar growth after winter dormancy
Source: Nat Commun. 2021 Feb 18;12:1123. doi: 10.1038/s41467-021-21449-0 (PMC7893051; doi:10.1038/s41467-021-21449-0)
Supplement: Supplementary file 4 — Description of Additional Supplementary Files [file 41467_2021_21449_MOESM4_ESM.pdf]

Descriptions of Additional Supplementary Files

Supplementary Data 1. Up-regulated genes in EBB3-RNAi apices during 10WSD

Supplementary Data 2. Up-regulated genes in EBB3-RNAi apices during 5WC

Supplementary Data 3. Up-regulated genes in EBB3-RNAi apices during 2WL

Supplementary Data 4. Down-regulated genes in EBB3-RNAi apices during 10WSD

Supplementary Data 5. Down-regulated genes in EBB3-RNAi apices during 2WC

Supplementary Data 6. Down-regulated genes in EBB3-RNAi apices during 5WC

Supplementary Data 7. Down-regulated genes in EBB3-RNAi apices during 2WLD

Supplementary Data 8. Common DEGs in the DML and EBB3 down-regulated transgenics

Supplementary Data 9. Gene ontology categories represented in the common DEGs in the DML and EBB3 down-regulated transgenics
