# Supplementary material for: EARLY BUD-BREAK 1 and EARLY BUD-BREAK 3 control resumption of poplar growth after winter dormancy
Source: Nat Commun. 2021 Feb 18;12:1123. doi: 10.1038/s41467-021-21449-0 (PMC7893051; doi:10.1038/s41467-021-21449-0)
Supplement: Supplementary file 2 — Peer Review [file 41467_2021_21449_MOESM2_ESM.pdf]

Reviewers' comments:

Reviewer #1 (Remarks to the Author):

The paper: “The EARLY BUD-BREAK regulatory module in Populus” addresses the molecular control of bud break in the seasonal growth, using the model tree hybrid aspen. As the authors stand, bud-break control is an environmental and economically important process where there is still a lot to know. There have been described two transcription factors involved in this activity, but they were so far not connected and a controlling mechanism was not discovered. In this work, the authors present a new regulatory module that controls this process, involving EBB1, SVL and EBB3, a newly discovered transcription factor. They use solid genetic, physiological and molecular analysis, still scarce in the study of this complex process in perennial tree species, to support their conclusions. The manuscript is well written, concise and provides novel and very interesting information regarding bud-break regulation of seasonal growth in a model tree species. The experiments are well conducted and interpreted. The work, in my view, has wide general interest and important applications.

I have several comments:

- Lines 38-41: In the introduction, related with the clock daylength sensing, there is a recent work missed. “LHY2 Integrates Night-Length Information to Determine Timing of Poplar Photoperiodic Growth” José M. Ramos-Sánchez et al., Current Biology, Vol. 29, Issue 14, p2402–2406.e4

- In figure 2, and paragraph beginning in line 109 “EBB3 is PtERF113 and its overexpression leads to early bud-break while its suppression causes a delay in bud-break”. It will be good to explain the use of two genotypes and if it is most likely that they behave similar, even though there is always the right control.

- In figure 3D and lines 154-158, “Indeed, the repressive mark H3K27me3 was significantly decreased at the EBB3 locus after exposure to low temperature that promotes dormancy release (Fig. 3D). The temporal correlation between decrease in H3K27me3 repressive marks in the EBB3 gene and its upregulation after low-temperature exposure (Fig. 3C) strongly suggest that H3K27me3 modification plays role in EBB3 regulation during dormancy release”. It should be better discussed the dynamics of H3K27me3 and why in 0W is always lower that in 4WC, if the expression of EBB3 goes down after bud break?

- Related with the epigenetic control of genes involved in bud break, a work describing a DEMETHYLASE involved in bud break in poplar should be commented. “Chilling-responsive DEMETER-LIKE DNA demethylase mediates in poplar bud break” Conde et al., Plant, Cell and Environment (2017) 40, 2236–2249. Moreover, the transcriptome studied in this work may be compared with the ones presented here.

- Figure 5: From SVL and ABA to EBB3, directly? Dashed lines?

- Related with the low temperature control of this regulatory module, it should be better described or in figure 7 substituted for a dashed arrow.

In summary, I consider that this paper has new and important data to be published in nature communications.

Reviewer #2 (Remarks to the Author):

The manuscript by Azeez and colleagues reports on the characterization of the regulatory module which includes two previously described transcription factors (EBB1 and SVL) and a newly identified transcription factor EBB3. This study ties together and further extends the work performed by Busov and Bhalearao labs in the last few years. They previously reported that a poplar AP2/ERF transcription factor EBB1 acted as the activator of budbreak, whilst SVL acted as a negative regulator of budbreak in the temperature and photoperiod-mediated phenology, through ABA and GA pathways and the downstream target TCP18. The conservation of key players (EBB1 similar to Arabidopsis meristem regulator DORNROSCHEN, SVL to floral repressor SVP and TCP18 to branching regulator BRC1) showed that conserved elements assembled in regulatory modules control vegetative/reproductive phenology and architecture in diverse plants, with implications in breeding and biotechnology of forest and fruit trees for the changing climate.

In this study, the authors continue the study of the molecular regulation underlying the mechanistic basis of budbreak, to link the cold temperature effects with activation of the cell cycle. They characterize the EBB3 gene, which activates budbreak, and integrate it in a regulatory module with EBB1 and SVL, connecting the hormonal regulation of dormancy and budbreak with cell cycle regulation. The work is thorough and convincing, the topic is timely and of interest, but some mistakes crept in and additional clarification and controls are needed.

1. Firstly, the authors discover another early budbreak mutant in the activation tagged poplar population and identify 2 candidate genes.

Line 97-98, precocious bud-break that was at least one week earlier… discrepancy with Figure 1D, where the average difference seems to be <6 days.

Line 102-103, Potri.012G108400 was 7.8 Kb downstream and Potri012G108500 2.4 Kb upstream of the activation tag insertion… Discrepancy with Figure 1C, in which Potri.012G108400 appears 7.8 Kb upstream and Potri012G108500 2.4 Kb downstream of the activation tag.

Figure 1A, it is unclear what is indicated by arrows.

Please clarify how many plants were used. At least 10 ramets were used for bud-break analysis (Figure 1 legend, line 730), were they from one or multiple clones?

The plants were propagated in tissue culture (line 339 on), then established in the glasshouse and subjected to manipulative dormancy experiment at the very young age. It is important to determine if older plants retain the early budbreak phenotype, for which monitoring for at least two seasons would be needed. Was such monitoring performed at least on the original ebb3D mutant plant? Similarly, all other transgenic lines are evaluated only at this early stage and not monitored over a longer period of time. This may be an issue, as some literature reports suggest that the dormancy-related phenotypes may change over years, e.g. in plants expressing SVP-like DAM genes.

2. Secondly, overexpression and RNAi experiments confirmed that EBB3 (another AP2/ERF) was the underlying cause for the observed mutant phenotype, acting as a positive regulator of budbreak.

Line 113, approximately 15 independent lines… how many for each gene (only 2 genes, so no need to approximate)

I might have missed it, but there seems to be no description or reference for the method for transformation of Agrobacterium C58 carrying overexpression constructs into WT-717 background. Please indicate if the same as reference 50. Also, was there a specific reason for transforming overexpression constructs into WT-717 and RNAi constructs into T89? Figures 2C and 2F suggest a big

difference in Days to budbreak between these WT backgrounds. If the conditions were different, that needs to be described in figure legend. Also please indicate n for the graphs showing the Time to budbreak.

3. The authors then interrogate expression of EBB3 to further support its role in activation of budbreak. Please describe the sampling of WT-717 tissues for Figure 3A - actively growing, so it is spring and therefore comparable with Mar or Apr in Figure 3B expression in wild aspen? I assume that tips are actively growing in both months, but the expression declines after March. Figure 3B, please indicate if these were apical or axillary buds, also the age of plants for panels A-C. It is unclear what is presented in Figure 3C, WT-717 or WT-T89 clones or something else – please clarify.

The upregulation over winter and during cold treatment supports the statement that EBB3 expression may be regulated by low temperature signals (line 135), however the expression peaks in March and at 2WLD treatment at warm temperatures – this needs to be discussed.

4. Next, the authors probe for a single histone repressive mark, H3K27me3, to investigate the epigenetic regulation of EBB3 induction. Please include appropriate negative controls from the ChIP-seq experiment, e.g. a couple of loci not induced by cold.

5. The authors move on to build the regulatory network, presenting several lines of evidence that EBB1 regulates SVL expression (opposing expression in buds and SVL expression in transgenic lines with altered EBB1 expression) and EMSA showed direct binding to the GCC-box in the SVL promoter. The same approach was used to include EBB3 in the common regulatory module, downstream from SVL and ABA, followed by transcriptomic and EMSA experiments placing CYCD3.1 as a direct EBB3 target. This part is clearly and convincingly presented.

Line 208-209, clarify last sentence, please

Other comments:

A few mistakes in Introduction, e.g. italics for genes and species
Line 40, CONSTANS not CONSTANT
Line 66, DAM genes, italics for DAM
Line 66, rephrase to clarify FLC is not a DAM gene
Line 73, another MADS-box gene with homology to SVP…, italics for SVP, italics for SVL
Line 80 EBB1 gene encoding a transcription factor…
Line 81, Arabidopsis italics please
Line 87, delete ‘an’

Reviewer #3 (Remarks to the Author):

Azeez et al. identified an AP2/ERF transcription factor, called EBB3, controlling bud outgrowth in poplar. The early bud-break of the ebb3D mutant was observed in field experiments and under controlled growth conditions. The EBB3 gene shows high expression in actively growing shoot apices and is downregulated at post-bud break. The expression pattern suggested that EBB3 plays a role during bud break; its expression is induced by low temperature, a condition which leads to the release from dormancy.

Authors suggest that EBB3 regulates bud break jointly with EBB1 and SVL, two genes previously identified to affect the process. Experimental evidence indicated that EBB1 is upstream of SVL

(negative regulation) and EBB3 (positive regulation, perhaps indirectly via SVL). However, binding of EBB1 to the GCC-box present in the SVL promoter was only demonstrated by EMSA (i.e., in vitro). This experiment cannot reveal whether EBB1 indeed binds to the SVL promoter in planta and this is a major limitation of the work currently presented. It is not surprising that EBB1 binds to the GCC box as binding to GCC-boxes is a generally known characteristic of AP2/ERF transcription factors. Most likely, other poplar AP2/ERF transcription factors (and even AP2/ERF transcription factors from other plants) would do the same in an EMSA experiment. Furthermore, EMSA experiments do not allow to determine under which in planta condition(s) EBB1 binds to the SVL promoter which, however, would be an important information to consolidate the model proposed by the authors. Authors should perform chromatin immunoprecipitation experiments. As poplar transformation is straightforward, such experiments can indeed be performed, e.g. with transgenic poplar plants expressing an EBB1-tag fusion from the native EBB1 promoter.

Considering the above, the statement given as a header in line 159 on page 7 (´EBB1 directly suppresses SVL expression´) is not solidly validated.

Authors provide data that indicate that EBB3 does not bind to the SVL promoter, however, again only EMSA was used to test for binding.

Overall, I find the two EMSA experiments shown in Fig. S3 and Fig. S4C difficult to understand, which may be due to the fact that the free DNA area has been cut away from both figures. Also, the labelling of two figures can be improved. What is the difference between lanes 4, 5, 6, and 7 in each of them?

In line 221, the authors state the EBB3 directly regulates expression of CYCD3;1. Also this statement is not validated by the experiments provided. The authors showed that EBB3 binds in vitro to the promoter of CYCD3;1. Again, in planta data are missing. Authors have the experience to perform ChIP experiments as shown in the chapter where epigenetic studies were performed

Overall, the model proposed by the authors is appealing; however, in the opinion of this reviewer, the current data are not solid enough to firmly prove the regulatory interactions proposed.

Other points:
• Writing of AP2-ERF vs. AP2/ERF: consistent writing (AP2/ERF) should be used throughout the manuscript.
• Lines 199/200: authors mention that ABA positively regulates expression of SVL, and that SVL positively regulates ABA biosynthesis and signaling genes, ´thus forming a feedback loop´. I think the authors want to say it is a positive feedback loop; it would be good to mention this.
• Lines 385/386 and following: authors describe the EMSA experiment performed for EBB1/SVL, but no information is given for the EBB3/SVL EMSA. Also, note that in the legend to Fig. S3 authors speak about EBB1 protein extract (or EBB1 protein) while in fact proteins extracted from protoplasts expressing the HA-tagged EBB1 protein where employed. This should also be clearly mentioned.
• The protoplast experiments were performed using protoplasts from Arabidopsis thaliana and not poplar. This information should be added to the manuscript.

Reviewers' comments:

Reviewer #1 (Remarks to the Author):
The paper: “The EARLY BUD-BREAK regulatory module in Populus” addresses the molecular control of bud break in the seasonal growth, using the model tree hybrid aspen. As the authors stand, bud-break control is an environmental and economically important process where there is still a lot to know. There have been described two transcription factors involved in this activity, but they were so far not connected and a controlling mechanism was not discovered. In this work, the authors present a new regulatory module that controls this process, involving EBB1, SVL, and EBB3, a newly discovered transcription factor. They use solid genetic, physiological, and molecular analysis, still scarce in the study of this complex process in perennial tree species, to support their conclusions. The manuscript is well written, concise, and provides novel and very interesting information regarding bud-break regulation of seasonal growth in a model tree species. The experiments are well conducted and interpreted. The work, in my view, has wide general interest and important applications.
In summary, I consider that this paper has new and important data to be published in nature communications.

I have several comments:
- Lines 38-41: In the introduction, related with the clock daylength sensing, there is a recent work missed. “LHY2 Integrates Night-Length Information to Determine Timing of Poplar Photoperiodic Growth” José M. Ramos-Sánchez et al., Current Biology, Vol. 29, Issue 14, p2402–2406.e4

Response: Thank you for pointing out this missing recent information. We have included this information in the revised manuscript.
Revised sentence: In poplar, the clock daylength/nightlength sensing components LHY1 (LATE ELONGATED HYPOCOTYL 1), LHY2, GI (GIGANTEA) and CO1/2 (CONSTANS) regulate *FT2*...(line 40-43)

- In figure 2, and paragraph beginning in line 109 “EBB3 is PtERF113 and its overexpression leads to early bud-break while its suppression causes a delay in bud-break”. It will be good to explain the use of two genotypes and if it is most likely that they behave similar, even though there is always the right control.

Response: The two genotypes were used because the overexpression and RNAi experiments were performed in two different laboratories using different genotypes. As the reviewer rightfully points out, we used the same conditions for the different dormancy induction

treatments and the proper controls of respective genotypes for each experiment. We believe that the fact that the outcomes from EBB3 functional alterations are consistent across different genotypes makes the conclusions from these experiments even stronger.

- In figure 3D and lines 154-158, "Indeed, the repressive mark H3K27me3 was significantly decreased at the EBB3 locus after exposure to low temperature that promotes dormancy release (Fig. 3D). The temporal correlation between decrease in H3K27me3 repressive marks in the EBB3 gene and its upregulation after low-temperature exposure (Fig. 3C) strongly suggest that H3K27me3 modification plays role in EBB3 regulation during dormancy release". It should be better discussed the dynamics of H3K27me3 and why in 0W is always lower that in 4WC, if the expression of EBB3 goes down after bud break?

Response: This is a very interesting question and we would have loved to elaborate on the topic in the paper but still very speculative as lacking solid experimental data and thus preferred to not discuss the issue in detail. We believe that the genes in the module, including EBB3, are under complex regulatory mechanisms including but not limited to chromatin modifications. Thus, the accumulation of repressive marks during dormancy establishment is likely the most important factor in EBB3 regulation during transition to dormancy. The removal of these marks by low temperature makes the gene more accessible but the gene is still under the regulation of other transcription factors. For example, transcription activators may upregulate the gene prior and at bud-break but repressors downregulate the gene past bud-break. However, what are these transcription factors and their signaling pathways are still unclear, and thus the speculative nature of this discussion.

- Related with the epigenetic control of genes involved in bud break, a work describing a DEMETHYLASE involved in bud break in poplar should be commented. "Chilling-responsive DEMETER-LIKE DNA demethylase mediates in poplar bud break" Conde et al., Plant, Cell and Environment (2017) 40, 2236–2249. Moreover, the transcriptome studied in this work may be compared with the ones presented here.

Response: We agree with the reviewer and apologize for not including this important information. We have included this work and comparison of two transcriptomics data in the revised manuscript.
Revised version of the manuscript (lines 313-323): The importance of epigenetic changes in regulation of low temperature response and bud-break was also recently demonstrated by the role of a poplar DEMETER-LIKE 10 (DML), a DNA methylase plays in regulation of bud-break. To find potential commonalities in the mechanisms governed by EBB3 and DML during bud-break, we compared the differentially expressed genes (DEGs) in EBB3 and DML knocked-down... (see text for full information and the associated data)

- Figure 5: From SVL and ABA to EBB3, directly? Dashed lines?

Response: We agree. Now in the revised Figure 7 (Model for bud-break), SVL-ABA to EBB3 represented by the dashed line, indicating an indirect connection.

- Related with the low temperature control of this regulatory module, it should be better described or in figure 7 substituted for a dashed arrow.
Response: Revised Figure 7 (Model for bud-break), low temperature regulated EBB1 is now represented by the dashed arrow.

Reviewer # 2
The manuscript by Azeez and colleagues reports on the characterization of the regulatory module which includes two previously described transcription factors (EBB1 and SVL) and a newly identified transcription factor EBB3. This study ties together and further extends the work performed by Busov and Bhalearao labs in the last few years. They previously reported that a poplar AP2/ERF transcription factor EBB1 acted as the activator of budbreak, whilst SVL acted as a negative regulator of budbreak in the temperature and photoperiod-mediated phenology, through ABA and GA pathways and the downstream target TCP18. The conservation of key players (EBB1 similar to Arabidopsis meristem regulator DORNROSCHEN, SVL to floral repressor SVP and TCP18 to branching regulator BRC1) showed that conserved elements assembled in regulatory modules control vegetative/reproductive phenology and architecture in diverse plants, with implications in breeding and biotechnology of forest and fruit trees for the changing climate. In this study, the authors continue the study of the molecular regulation underlying the mechanistic basis of budbreak, to link the cold temperature effects with activation of the cell cycle. They characterize the EBB3 gene, which activates budbreak, and integrate it in a regulatory module with EBB1 and SVL, connecting the hormonal regulation of dormancy and budbreak with cell cycle regulation.
The work is thorough and convincing, the topic is timely and of interest, but some mistakes crept in and additional clarification and controls are needed.

1. Firstly, the authors discover another early budbreak mutant in the activation tagged poplar population and identify 2 candidate genes.
Line 97-98, precocious bud-break that was at least one week earlier… discrepancy with Figure 1D, where the average difference seems to be <6 days.

Response: Thank you for pointing this out. We have corrected this now and have included this information in the revised manuscript.
The revised sentence: Mutant plants showed precocious bud-break that was around 6 days earlier as compared to WT-717 (Results section, line 98-100, Fig. 1D).

Line 102-103, Potri.012G108400 was 7.8 Kb downstream and Potri012G108500 2.4 Kb upstream of the activation tag insertion... Discrepancy with Figure 1C, in which Potri.012G108400 appears 7.8 Kb upstream and Potri012G108500 2.4 Kb downstream of the activation tag.

Response: We have corrected this now and have included the suggested information in the revised manuscript.
The revised sentence: Potri.012G108400 was 7.8Kb upstream and Potri.012G108500 2.4Kb downstream of the activation tag insertion (Results section, lines 104-105, Fig. 1C).

Figure 1A, it is unclear what is indicated by arrows.

Response: We have clarified this now and have included the requested information in the Figure 1 legend.
The revised legends of Figure 1A (lines 784-786): *ebb3D* poplar mutant plants show early bud-break ... arrows point to two *ebb3D* ramets that show early bud-break compared to the WT-717 plants and other neighboring activation tagging events.

Please clarify how many plants were used. At least 10 ramets were used for bud-break analysis (Figure 1 legend, line 730), were they from one or multiple clones?

Response: We have clarified this now and have included this information in Figure 1 legend.
The revised legends of Figure 1 (line 791): At least 10 plants of the *ebb3D* mutant and WT-717 genotypes were used in the bud-break analysis.

The plants were propagated in tissue culture (line 339 on), then established in the glasshouse and subjected to manipulative dormancy experiment at the very young age. It is important to determine if older plants retain the early budbreak phenotype, for which monitoring for at least two seasons would be needed. Was such monitoring performed at least on the original ebb3D mutant plant? Similarly, all other transgenic lines are evaluated only at this early stage and not monitored over a longer period of time. This may be an issue, as some literature reports suggest that the dormancy-related phenotypes may change over years, e.g. in plants expressing SVP-like DAM genes.

Response: We have identified both early bud break mutants (ebb1D and ebb3D) in older plants under field conditions. We then recapitulated the phenotype of both mutants in plants under growth chamber conditions in young plants. The phenotypes (e.g. early bud-break) in both older and younger plants were the same. This is shown in Figures 1 in both our earlier PNAS EBB1 paper and the current paper. We thus believe that phenotypes are maintained in plants irrespective of age as well as between natural and controlled conditions.

To make this clearer, we have now wording in the Methods section clearly stating the age of the experimental plants as follows (lines348-350): "The *EARLY BUD-BREAK3D* (*ebb3D*) mutant was discovered in a field trial with 2-year-old plants...."

To validate the phenotypes, the candidate genes were retransformed into hybrid poplar again and these were subjected to phenotypic analysis in the growth chamber, which allows much more rigorous analysis as well as the comparison between various mutants.

The size of the older plants exceeds the size of the growth chambers and thus experiments with older (bigger) plants are not feasible. Phenotyping under field conditions with older plants, as we have done with the mutants' screening, is extraordinarily difficult especially since field testing with transgenic plants is under strict regulations and thus permits, particularly with perennial trees are hard or in some cases impossible to obtain.

Given the correspondence of the phenotypic analyses we have observed with the older mutant plants and younger recapitulation transgenics under both field and controlled

growth chamber conditions leads us to suggest that phenotyping in older plants will likely not significantly add to the paper.

2. Secondly, overexpression and RNAi experiments confirmed that EBB3 (another AP2/ERF) was the underlying cause for the observed mutant phenotype, acting as a positive regulator of budbreak.
Line 113, approximately 15 independent lines … how many for each gene (only 2 genes, so no need to approximate)

Response: We have clarified this now and have included the exact number of lines recovered for each gene.
The revised sentence (lines 115-117): A total of 13 Potri.012G108400/RPL34e and 18 Potri.012G108500/PtERF113 overexpressing independent transgenic events were recovered and three lines with high overexpression (Fig. 2B & Fig. S2A) ...

I might have missed it, but there seems to be no description or reference for the method for transformation of Agrobacterium C58 carrying overexpression constructs into WT-717 background. Please indicate if the same as reference 50.

Response: We missed one reference and now have added a sentence in the methods.
The revised sentence (lines 390-391): The transformation into WT-T89 and WT-717 plants were done as previously described (added both references).

Also, was there a specific reason for transforming overexpression constructs into WT-717 and RNAi constructs into T89? Figures 2C and 2F suggest a big difference in Days to budbreak between these WT backgrounds. If the conditions were different, that needs to be described in figure legend. Also please indicate n for the graphs showing the Time to budbreak.

Response: The reason for using the two different genotypes is that the overexpression and RNAi studies were performed in two different laboratories, which use the different genotypes in their research. As the reviewer rightfully points out, we used the same conditions for the different dormancy induction treatments and the proper controls of respective genotypes for each experiment. We believe that the fact that the outcomes from EBB3 functional alterations are consistent across different genotypes makes the conclusions from these experiments even stronger.

3. The authors then interrogate expression of EBB3 to further support its role in activation of budbreak. Please describe the sampling of WT-717 tissues for Figure 3A - actively growing, so it is spring and therefore comparable with Mar or Apr in Figure 3B expression in wild aspen? I assume that tips are actively growing in both months, but the expression declines after March. Figure 3B, please indicate if these were apical or axillary buds, also the age of plants for panels A-C.

Response: Although in essence, the reviewer is correct that the apex and Mar-Apr samples from Fig. 3A and B respectively are developmentally equivalent, there are important differences. First, tissues for Fig. 3A were obtained from greenhouse grown plants that

have never gone through a dormancy cycle. This allowed us to collect samples uniformly at the same time under the same conditions. The expression of the gene in the apex is high relative to the other tissues. The gene is generally expressed at very low levels and Fig. 3A reflects relative expression to each other not as an absolute amount that can be compared to other experiments (e.g., Fig. 3B). We apologize for this have now provided detailed information of each sampling hoping that this dispels any previous confusions.
We have clarified this now and have included the sampling procedure in the Methods section (lines 391-398).
Revised sentence: For expression analysis during the dormancy cycle, we collected vegetative apical buds from small branches of three individual wild aspen (*Populus tremuloides*, approximately 20 years-old) trees for each month of the dormancy period (September to April). Whole buds were immediately frozen in liquid nitrogen. For tissue-specific expression analysis, different tissue samples were collected from greenhouse grown four months old WT-717 plants and stored at −80°C until processed.

It is unclear what is presented in Figure 3C, WT-717 or WT-T89 clones or something else – please clarify.

Response: Figure 3C represents expression during the growth-dormancy cycle of T89 genotype under controlled growth chamber conditions. We have accordingly revised the legend to clarify this as follows: (line 851-852): Relative expression of *EBB3* in WT-T89 plants under manipulative dormancy treatments in controlled growth chamber conditions."

We have also revised the sentence in the text referring to this figure as follows (lines 141-142): "To more precisely correlate the expression of *EBB3* ..., we exposed poplar plants (WT-T89) to a regime of short days (10WSD) to induce growth cessation and dormancy."

The upregulation over winter and during cold treatment supports the statement that EBB3 expression may be regulated by low temperature signals (line 135), however the expression peaks in March and at 2WLD treatment at warm temperatures – this needs to be discussed.

Response: As we have mentioned in a previous response the regulation of EBB3 and the other genes in the module is complex. We show clear evidence that chromatin repressive marks and their temporal accumulation during SD treatment and removal in cold are the most likely cause for the low and high expression of the gene during these periods. However, the removal of the repressive marks only makes the gene accessible to transcription regulators. Once accessible, the gene is then activated by either or both the increasing transcription activator or declining repressor and thus the peak of the expression in March/2WLD.

4. Next, the authors probe for a single histone repressive mark, H3K27me3, to investigate the epigenetic regulation of EBB3 induction. Please include appropriate negative controls from the ChIP-seq experiment, e.g. a couple of loci not induced by cold.

Response: We have previously performed H3K27me3 analysis of SVL (a gene not induced by low temperature) from the same material as used here (Singh et al., 2019; Nature Comm). In contrast with EBB3, low temperature had no effect on H3K27me3 levels at the

SVL locus suggesting that alteration of H3K27me3 levels observed at EBB3 is specific response and not a general genome wide low temperature modulation of H3K37me3.

Line 208-209, clarify last sentence, please

Response: We have modified the sentence now as suggested.

The revised sentence is as follows (lines 218-220): Thus, these data indicate that EBB3 is a target of ABA signaling downstream of the SVL/ABA feedforward loop during dormancy release and bud-break.

Other comments:

A few mistakes in Introduction, e.g. italics for genes and species
Line 40, CONSTANS not CONSTANT
Line 66, DAM genes, italics for DAM
Line 66, rephrase to clarify FLC is not a DAM gene
Line 73, another MADS-box gene with homology to SVP … , italics for SVP, italics for SVL
Line 80 EBB1 gene encoding a transcription factor…
Line 81, Arabidopsis italics please
Line 87, delete 'an'

Response: Thanks for pointing out these, we have corrected the mistakes (highlighted in the text).

Reviewer #3 (Remarks to the Author):
Azeez et al. identified an AP2/ERF transcription factor, called EBB3, controlling bud outgrowth in poplar. The early bud-break of the ebb3D mutant was observed in field experiments and under controlled growth conditions. The EBB3 gene shows high expression in actively growing shoot apices and is downregulated at post-bud break. The expression pattern suggested that EBB3 plays a role during bud break; its expression is induced by low temperature, a condition which leads to the release from dormancy. Authors suggest that EBB3 regulates bud break jointly with EBB1 and SVL, two genes previously identified to affect the process. Experimental evidence indicated that EBB1 is upstream of SVL (negative regulation) and EBB3 (positive regulation, perhaps indirectly via SVL). However, binding of EBB1 to the GCC-box present in the SVL promoter was only demonstrated by EMSA (i.e., in vitro). This experiment cannot reveal whether EBB1 indeed binds to the SVL promoter in planta and this is a major limitation of the work currently presented. It is not surprising that EBB1 binds to the GCC box as binding to GCCboxes is a generally known characteristic of AP2/ERF transcription factors. Most likely, other poplar AP2/ERF transcription factors (and even AP2/ERF transcription factors from other plants) would do the same in an EMSA experiment. Furthermore, EMSA experiments do not allow to determine under which in planta condition(s) EBB1 binds to the SVL promoter which, however, would be an important information to consolidate the model proposed by the authors. Authors should perform chromatin immunoprecipitation experiments. As poplar transformation is straightforward, such experiments can indeed be performed, e.g. with transgenic poplar plants expressing an EBB1-tag fusion from the native EBB1 promoter.
Considering the above, the statement given as a header in line 159 on page 7 (´EBB1 directly suppresses SVL expression´) is not solidly validated.

Response: We agree with the reviewer. To address reviewer's concern, we performed ChIP-qPCR experiments with poplar protoplasts (see details in the manuscript) to confirm the EBB1 binding in the SVL promoter in planta. Consistent with our in vitro binding assay, the ChIP-qPCR results showed strong binding of EBB1 to the promoter region of *SVL* containing the GCC box (Fig. 4D).
To address reviewer's concern about AP2/ERF general binging to GCC-box in EMSA experiments, we have checked the EBB3 (AP2/ERF) binding with a different GCC-box and it does not show any binding (Fig. S4C). Note that the oligos that are used in these experiments include contextual sequence around the box which is different in the different regions. These contextual sequences are very important for the binding of the transcription factors as we show here.

Authors provide data that indicate that EBB3 does not bind to the SVL promoter, however, again only EMSA was used to test for binding.

Response: As with the previous comment, we agree with the reviewer. We therefore performed ChIP-qPCR experiments that tested EBB3 binding to *SVL* promoter in poplar protoplasts. Consistent with our in vitro binding assay, no evidence of EBB3 binding to the regulatory region of the *SVL* was detected by these experiments (Fig. S4D)

Overall, I find the two EMSA experiments shown in Fig. S3 and Fig. S4C difficult to understand, which may be due to the fact that the free DNA area has been cut away from both figures. Also, the labeling of the two figures can be improved. What is the difference between lanes 4, 5, 6, and 7 in each of them?

Response: Thanks for pointing this out. We have updated the labeling to make it clearer to understand. Regarding the free DNA issue, we used two sizes of probes in these EMSA assays. Because of the differences in size, the smaller one was always running out of the gel and thus not present. We preferred to run everything in the same gel to make the results more consistent and comparable. Now all of these results are further validated with ChIP-qPCR experiments.

In line 221, the authors state the EBB3 directly regulates expression of CYCD3;1. Also this statement is not validated by the experiments provided. The authors showed that EBB3 binds in vitro to the promoter of CYCD3;1. Again, in planta data are missing. Authors have the experience to perform ChIP experiments as shown in the chapter where epigenetic studies were performed

Response: As with the previous comments regarding in vivo binding, we agree with the reviewer. We therefore performed ChIP-qPCR experiments that tested EBB3 binding to GCC-box in the CYCD3.1 promoter in poplar protoplasts. We find clear evidence of EBB3 binding in the *CYCD3.1* promoter (Fig. 6C, lines 244-248).

Overall, the model proposed by the authors is appealing; however, in the opinion of this reviewer, the current data are not solid enough to firmly prove the regulatory interactions proposed.

Response: We hope that the additional data provided as requested by the reviewer are now providing more solid support to the proposed model.

Other points:

- Writing of AP2-ERF vs. AP2/ERF: consistent writing (AP2/ERF) should be used throughout the manuscript.

Response: Changes made in the revised manuscript.

- Lines 199/200: authors mention that ABA positively regulates expression of SVL, and that SVL positively regulates ABA biosynthesis and signaling genes, ´thus forming a feedback loop´. I think the authors want to say it is a positive feedback loop; it would be good to mention this.

Response: Changes made in the revised manuscript.

- Lines 385/386 and following: authors describe the EMSA experiment performed for EBB1/SVL, but no information is given for the EBB3/SVL EMSA. Also, note that in the legend to Fig. S3 authors speak about EBB1 protein extract (or EBB1 protein) while in fact proteins extracted from protoplasts expressing the HA-tagged EBB1 protein where employed. This should also be clearly mentioned.

Response: Changes made in the revised manuscript (lines 456-457)

- The protoplast experiments were performed using protoplasts from Arabidopsis thaliana and not poplar. This information should be added to the manuscript.

Response: Changes made in the revised manuscript (Line 456-457)

Reviewer #1 (Remarks to the Author):

Dear Authors,
Now I consider that my concerns have been solve, only one mistake
in this revised version: DML is a DNA demethylase

"Revised version of the manuscript (lines 313-323): The importance of epigenetic changes in regulation of low temperature response and bud-break was also recently demonstrated by the role of a poplar DEMETER-LIKE 10 (DML), a DNA methylase plays in regulation of bud-break. To find potential commonalities in the mechanisms governed by EBB3 and DML during bud-break, we compared the differentially expressed genes (DEGs) in EBB3 and DML knocked-down... (see text for full information and the associated data)"

Other than this, I consider the manuscript ready to be published, congratulation

Reviewer #2 (Remarks to the Author):

The authors have addressed my concerns. The addition of new results (protoplast binding assays) to support the claims and expand the SVL-ABA model has further improved the manuscript. There is still some room for improvement, but this should be easy to address.

The main text and figures – could be better organized so that the Results subsections correspond to Figures (under correct subheadings). This applies to Figures 4-6. For example, Fig. 4E, it would make more sense to move it to Figure 5, as it is described under the paragraph related to Fig. 5, focusing on EBB3 acting downstream from EBB1 and SVL.

RNA-seq – discrepancies in numbers reported in text L227-228 and Supplementary Table 2, compared to the Venn diagram in Figure 6A. Please update with corrected numbers. Fig. 6A legend – please explain panel A. Are these downregulated transcripts, as per Datasets S5-S8? If yes, there are discrepancies for 10 WSD and 2 WLD when compared to Supplementary Table 2! Please read through carefully to avoid such oversights and errors.

Figure 6A, please point out where in the Venn diagram is the CycD transcript. Your current Fig 6A is just a diagram, it does not show that CYCD3.1 is differentially expressed, it just breaks down the numbers of significant DEGs between libraries. The identity of other DEGs commonly expressed between at least 2 datasets could easily be added, given they range between 0 and 2.

Figure 3, is there an explanation for low expression and low H3K27me3 at 0W? Any significance in a couple of peaks in a few hundred bp upstream and at the TSS (dormant and chilled compared to 0W)? I have just realized this was raised by another reviewer and the discussion would be speculative – while the conclusion is toned down, the low H3K27me3 in 0W needs some explanation, even if it is just pointing out that additional transcriptional regulatory mechanisms might be involved.

Method, plasmid constructs, please indicate the promoters driving the CDS, RNAi, and GFP- and HA-tagged fragments.

Minor
L74-75, Moser et al demonstrated loss of growth cessation and dormancy in apple, resulting from co-suppression of apple DAM1, recently published (doi.org/10.3389/fpls.2020.01003)
L107, compared
L126, order of figure panels in the text
L150, regulates
L179, refer to Fig. 4D for the GCC-box at -150 bp

Line 202-205, italics for SVL transgenic plants, same for SVL promoter in Fig. S4 legend.
L223, EBB3 is a member of the AP2/ERF TF family. So? As is, this sentence is not needed (or should lead into the justification of the RNA-seq experiment).
Page 22, L489, ChIP-seq experiment, what about 0W and 6WSD samples, also presented in Fig. 3D?

[Editor: Reviewer #2 provides his/her comments on your responses to Reviewer #3 previous suggestions in Remark to Editor section. Below is the summarized version of the comments. Please revise your manuscript accordingly.]

The main concern Reviewer #3 raised is the lack of in vivo confirmation of promoter binding. EMSA shows in vitro binding of EBB1 to SVL promoter, but it does not allow to determine under which in planta condition(s) EBB1 binds to the SVL promoter. Therefore, Reviewer #3 requested the authors perform chromatin immunoprecipitation experiments, using poplar transformation e.g. with transgenic poplar plants expressing an EBB1-tag fusion from the native EBB1 promoter.
For the GFP tagged EBB1 (and EBB3 for other similar tests), the authors amplify the coding regions, then recombine into pMDC83. In their methods, it was not made clear what was the promoter used in this vector. After checking the pMDC83 vector sequence available on-line, it seems to me that Gateway cloning places the EBB1/EBB3 coding regions between 35S and GFP. Reviewer #3 might have objected the use of a strong constitutive promoter rather than the native promoters.

Further, the authors use poplar protoplasts, followed by ChIP-PCRs of their predicted target. Therefore I am not sure that the experiment addresses the concerns raised by Reviewer #3, as it still does not allow to determine under which in planta condition(s) EBB1 binds to the SVL promoter.

However, the experiment the authors perform does provide another line of evidence that binding can occur (at least when lots of protein is introduced into protoplasts). Importantly, it is a result the authors were able to deliver in a timely fashion. The authors can perhaps rephrase, e.g. line187, thus the in vitro and in vivo binding assays indicate that EBB1 can directly bind the SVL promoter, and may be able to regulate its expression (instead of …clearly indicate that EBB1 directly binds to SVL promoter to regulate its expression). Similar toning down would be needed for other ChIP-PCR experiments, and the authors would need to discuss the lack of in planta confirmation of the proposed model.

Response to reviewer comments

Reviewer #1 (Remarks to the Author):

Dear Authors,
Now I consider that my concerns have been solve, only one mistake in this revised version: DML is a DNA demethylase

"Revised version of the manuscript (lines 313-323): The importance of epigenetic changes in regulation of low temperature response and bud-break was also recently demonstrated by the role of a poplar DEMETER-LIKE 10 (DML), a DNA methylase plays in regulation of bud-break. To find potential commonalities in the mechanisms governed by EBB3 and DML during bud-break, we compared the differentially expressed genes (DEGs) in EBB3 and DML knocked-down... (see text for full information and the associated data)"

Other than this, I consider the manuscript ready to be published, congratulation

Response: Thank you for pointing this out. We have corrected the error in the revised manuscript (lines: 318-322).

Reviewer #2 (Remarks to the Author):

The authors have addressed my concerns. The addition of new results (protoplast binding assays) to support the claims and expand the SVL-ABA model has further improved the manuscript. There is still some room for improvement, but this should be easy to address.

The main text and figures – could be better organized so that the Results subsections correspond to Figures (under correct subheadings). This applies to Figures 4-6. For example, Fig. 4E, it would make more sense to move it to Figure 5, as it is described under the paragraph related to Fig. 5, focusing on EBB3 acting downstream from EBB1 and SVL.

Response: We agree and have rearranged Figures 4 and 5 as suggested so now they correspond well to the figures as describe in the main text.

RNA-seq – discrepancies in numbers reported in text L227-228 and Supplementary Table 2, compared to the Venn diagram in Figure 6A. Please update with corrected numbers. Fig. 6A legend – please explain panel A. Are these downregulated transcripts, as per Datasets S5-S8? If yes, there are discrepancies for 10 WSD and 2 WLD when compared to Supplementary Table 2! Please read through carefully to avoid such oversights and errors.

Response: We have corrected the errors and included Figure 6A legend as follows:

The revised sentence (lines 226-228): "There were 29 differentially expressed genes after dormancy establishment (10WSD), 110 after 5-week cold treatment (5WC), and 1,162 at the…."

The revised legends of Figure 6A (line 936-939): (A) Number of common and unique downregulated genes in EBB3 RNAi under-expressing plants in different dormancy stages. The *Cyclin D3.1* (*CYCD3.1*) is the only downregulated gene in all dormancy stages. See Supplementary dataset 1 for more details.

Figure 6A, please point out where in the Venn diagram is the CycD transcript. Your current Fig 6A is just a diagram, it does not show that CYCD3.1 is differentially expressed, it just breaks down the numbers of significant DEGs between libraries. The identity of other DEGs commonly expressed between at least 2 datasets could easily be added, given they range between 0 and 2.

Response: As suggested, we have updated the Venn diagram and also labelled the CYCD3.1 in the figure to make it clearer to understand. The identity of all downregulates genes provided in the Supplementary dataset 1 (Dataset S5-8), and we also for clarity highlighted CYCD3.1 in green font at all time points (Dataset S5-8).

Figure 3, is there an explanation for low expression and low H3K27me3 at 0W? Any significance in a couple of peaks in a few hundred bp upstream and at the TSS (dormant and chilled compared to 0W)? I have just realized this was raised by another reviewer and the discussion would be speculative – while the conclusion is toned down, the low H3K27me3 in 0W needs some explanation, even if it is just pointing out that additional transcriptional regulatory mechanisms might be involved.

Response: We agree with the reviewer and have included the following text in the discussion to address this: "This correlation between repressive marks and expression is not perfect and indicates that EBB3 is under complex regulatory mechanism including but not limited to epigenetic modifications. For example, transcription activators may upregulate the gene prior and at bud-break but repressors downregulate the gene past bud-break. Thus, the summary of all data strongly implicates EBB3 in the regulation of bud-break in poplar and this may involve at least in part low temperature-responsive changes in *EBB3* expression via epigenetic changes."

Method, plasmid constructs, please indicate the promoters driving the CDS, RNAi, and GFP- and HA-tagged fragments.

Response: The requested changes were made in the revised manuscript.

Minor

L74-75, Moser et al demonstrated loss of growth cessation and dormancy in apple, resulting from co-suppression of apple DAM1, recently published (doi.org/10.3389/fpls.2020.01003)

L107, compared

L126, order of figure panels in the text

L150, regulates

L179, refer to Fig. 4D for the GCC-box at -150 bp
Line 202-205, italics for SVL transgenic plants, same for SVL promoter in Fig. S4 legend.
L223, EBB3 is a member of the AP2/ERF TF family. So? As is, this sentence is not needed (or should lead into the justification of the RNA-seq experiment).

Response: We have corrected the errors in the revised manuscript.

Page 22, L489, ChIP-seq experiment, what about 0W and 6WSD samples, also presented in Fig. 3D?

Response: We have added the information.

[Editor: Reviewer #2 provides his/her comments on your responses to Reviewer #3 previous suggestions in Remark to Editor section. Below is the summarized version of the comments. Please revise your manuscript accordingly.]

The main concern Reviewer #3 raised is the lack of in vivo confirmation of promoter binding. EMSA shows in vitro binding of EBB1 to SVL promoter, but it does not allow to determine under which in planta condition(s) EBB1 binds to the SVL promoter. Therefore, Reviewer #3 requested the authors perform chromatin immunoprecipitation experiments, using poplar transformation e.g. with transgenic poplar plants expressing an EBB1-tag fusion from the native EBB1 promoter.
For the GFP tagged EBB1 (and EBB3 for other similar tests), the authors amplify the coding regions, then recombine into pMDC83. In their methods, it was not made clear what was the promoter used in this vector. After checking the pMDC83 vector sequence available on-line, it seems to me that Gateway cloning places the EBB1/EBB3 coding regions between 35S and GFP. Reviewer #3 might have objected the use of a strong constitutive promoter rather than the native promoters.

Response: Using native or 35S promoter are two different approaches and each has pros and cons. The reviewer rightfully points out the merits of the approach using the native promoter. However, 35S promoter as well has been successfully and often used to identify target genes of transcription factors in Arabidopsis and other species including poplar and is a mainstream experimental approach to address this type of questions. Importantly, we draw our conclusions based on four independent methods including: EMSA, ChIP-qPCR, transgenics and expression experiments. All strongly suggest that EBB1 negatively and directly regulates SVL while EBB3 positively and directly regulates CYCD3.1.

Further, the authors use poplar protoplasts, followed by ChIP-PCRs of their predicted target. Therefore I am not sure that the experiment addresses the concerns raised by Reviewer #3, as it still does not allow to determine under which in planta condition(s) EBB1 binds to the SVL promoter.

Response: EBB1 and EBB3 are highly expressed in actively growing or competent to grow tissues. Protoplasts are derived from actively growing leaves and therefore approximate the

types of cells where these genes are expressed under in planta conditions. Again ChIP-PCR is one of three other independent methods used by us and importantly the alternative approach of EMSA assays, along with the transgenic and the expression data all converge on the same conclusion. Thus, while we agree with the reviewer that it would be interesting and informative to pinpoint the exact time, place, tissue and affinity of these interactions, these are somewhat out of scope of the focus of this manuscript.

However, the experiment the authors perform does provide another line of evidence that binding can occur (at least when lots of protein is introduced into protoplasts). Importantly, it is a result the authors were able to deliver in a timely fashion. The authors can perhaps rephrase, e.g. line187, thus the in vitro and in vivo binding assays indicate that EBB1 can directly bind the SVL promoter, and may be able to regulate its expression (instead of …clearly indicate that EBB1 directly binds to SVL promoter to regulate its expression).

Response: We take the point raised by the reviewer and agree with the reviewer's suggestion. Changes were made in the revised manuscript (lines: 187-188).

Similar toning down would be needed for other ChIP-PCR experiments, and the authors would need to discuss the lack of in planta confirmation of the proposed model.

Response: We have added language to the discussion to indicate the need for future in planta experiment and what these could add to the model as follows: "The identification of these regulatory interactions would benefit further from in planta analysis of their spatiotemporal specificity during dormancy-activity cycle."

Reviewer #2 (Remarks to the Author):

The authors have addressed my concerns, thank you.
